# A preliminary transcriptomic analysis of the orbitofrontal cortex of antisocial individuals

**DOI:** 10.1111/cns.14283

**Published:** 2023-06-02

**Authors:** Ignazio S. Piras, Giulia Braccagni, Matthew J. Huentelman, Marco Bortolato

**Affiliations:** ^1^ Neurogenomics Division Translational Genomics Research Institute (TGen) Phoenix Arizona USA; ^2^ Department of Pharmacology and Toxicology College of Pharmacy University of Utah Salt Lake City Utah USA

**Keywords:** antisocial personality disorder, conduct disorder, orbitofrontal cortex, transcriptomics

## Abstract

**Aims:**

Antisocial personality disorder (ASPD) and conduct disorder (CD) are characterized by a persistent pattern of violations of societal norms and others' rights. Ample evidence shows that the pathophysiology of these disorders is contributed by orbitofrontal cortex (OFC) alterations, yet the underlying molecular mechanisms remain elusive. To address this knowledge gap, we performed the first‐ever RNA sequencing study of postmortem OFC samples from subjects with a lifetime diagnosis of ASPD and/or CD.

**Methods:**

The transcriptomic profiles of OFC samples from subjects with ASPD and/or CD were compared to those of unaffected age‐matched controls (*n* = 9/group).

**Results:**

The OFC of ASPD/CD‐affected subjects displayed significant differences in the expression of 328 genes. Further gene‐ontology analyses revealed an extensive downregulation of excitatory neuron transcripts and upregulation of astrocyte transcripts. These alterations were paralleled by significant modifications in synaptic regulation and glutamatergic neurotransmission pathways.

**Conclusion:**

These preliminary findings suggest that ASPD and CD feature a complex array of functional deficits in the pyramidal neurons and astrocytes of the OFC. In turn, these aberrances may contribute to the reduced OFC connectivity observed in antisocial subjects. Future analyses on larger cohorts are needed to validate these results.

## Graphical abstract

We performed the first transcriptomic analysis of postmortem orbitofrontal cortex samples from subjects with antisocial personality disorder (ASPD) and/or conduct disorder (CD). Our results indicate that ASPD and/or CD are characterized by dysfunctions of synaptic regulation and neurotransmission in the pyramidal neurons of the orbitofrontal cortex. These findings point to molecular and functional substrates that may explain the connectivity deficits exhibited by the orbitofrontal cortex of antisocial subjects.

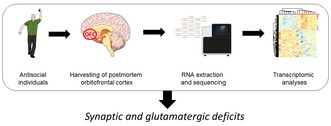

## 1 INTRODUCTION

Antisocial behavior is a persistent and pervasive pattern of disregard and infringement of societal norms, frequently resulting in damage to the rights of others as well as covert and overt hostility.^1^ From a clinical perspective, the two major diagnostic entities characterized by antisocial tendencies are conduct disorder (CD) in children and adolescents and antisocial personality disorder (ASPD) in adults. These disorders share similar diagnostic criteria, including failure to conform to rules and laws, deceitfulness, and physical aggression.^2^ The estimated lifetime prevalence of CD and ASPD in the general population is 9.5%^3^ and 4.3%,^4^ respectively, with approximately 40% of individuals with CD exhibiting persistent ASPD in adulthood.^5^ However, among incarcerated individuals, the prevalence rate of ASPD is estimated to rise to about 47%,^6^ reflecting the strong correlation between these disorders and criminal behavior.

Both ASPD and CD are characterized by a marked male preponderance, with male‐to‐female ratios ranging from 3:1 to 7:1.^7^, ^8^ These disorders frequently co‐occur with other psychiatric conditions, the most prevalent of which are substance use disorder (SUD) and depression (with comorbidity rates estimated at 90%–95% and 20%, respectively).^9^, ^10^ The neurobiological basis of ASPD and CD is a topic of ongoing investigations, with genetic and neuroimaging analyses providing insight into their ontogeny. A substantial body of evidence suggests that antisocial behavior is influenced by a broad constellation of genes, each of which likely contributes only a small portion to the overall phenotype.^11^ Given the well‐documented role of serotonin in modulating aggression,^12^ most studies have focused on genes related to this neurotransmitter system, particularly on *MAOA* (monoamine oxidase A), which encodes the primary enzyme responsible for its degradation.^13^ An extensive body of evidence points to this gene as a critical determinant of antisocial behavior.^14^ In addition, a recent meta‐analysis based on genome‐wide association studies (GWAS) in antisocial individuals has shown a significant association between *FOXP2* (transcription factor forkhead box protein P2) and antisocial behavior.^15^

Several lines of neuroimaging research have shown that the maladaptive social responses in antisocial behavior are contributed by structural and functional deficits of the orbitofrontal cortex (OFC)^16^ (for scholarly discussions of the role of the OFC in antisocial behavior, see refs. [17, 18]). Through its connectivity with limbic regions (and primarily the amygdala), this brain area regulates the interpretation of social cues and the enactment of appropriate emotional reactions.^19^, ^20^ OFC lesions lead to behavioral aberrances akin to those observed in antisocial individuals, including disinhibition, social inappropriateness, lack of concern with the consequences of one's action, and poor ability to recognize affective cues.^21^ In line with these premises, converging lines of evidence highlight that OFC dysfunctions play a pivotal role in the pathophysiology of ASPD^22^ and related personality traits, including aggression, disinhibition, impulsivity, lack of insight, and psychopathy.^23^, ^24^, ^25^, ^26^, ^27^ These advances notwithstanding, the neurochemical mechanisms whereby the OFC contributes to the ontogeny of antisocial behavior remain elusive. To address this knowledge gap, here we conducted the first‐ever transcriptomic analysis of postmortem OFC samples from individuals affected by either ASPD or CD in relation to age‐matched unaffected controls.

## 2 MATERIALS AND METHODS

### 2.1 Human brain collection and donor information

The postmortem OFC samples were previously used in another study.^28^ Tissues were obtained from the NIH NeuroBioBank (NBB) Brain and Tissue Repository (BTR) at the University of Pittsburgh. The right hemisphere of each brain was blocked coronally, immediately frozen, and stored at –80°C per the policies and procedures utilized by the BTRs participating in the NIH NBB. The right OFC (defined by the medial orbital gyrus) was harvested. White matter was avoided during sample acquisition using the gross anatomical features that distinguish it from gray matter. No gross pathological atrophy was identified in the OFC region of any subject.

The selection of the right OFC was driven by previous data showing that this region features selective volumetric reductions in ASB^24^ and is directly involved in anger processing.^29^ Consent was obtained according to legal provisions from the next of kin. A Medical Examiner evaluated manners of death (natural, accidental, or suicide). All subjects died suddenly and out‐of‐hospital, with no evidence of an agonal state. One individual (subject B7; see Table 2) died of suicide due to a gun wound in the mid‐pons, with no alterations found in any cortical region. Lifetime psychiatric diagnoses were established postmortem using a rigorous process, including (1) extensive medical record collection and review; (2) structured psychodiagnostic interviews (SCID‐5‐RV and SCID‐5‐PD) with the next‐of‐kin or another knowledgeable informant; and (3) a postmortem diagnostic conference staffed by adult, child, and geriatric psychiatrists, clinical psychologists, and other senior psychiatric clinicians, which generates ICD‐10‐CM diagnoses for all subjects.

The absence of a psychiatric diagnosis was confirmed in unaffected control subjects using the same approach. The analysis was performed in postmortem OFC tissue (21.68 ± 3.80 mg/sample) and associated clinical data, including age, sex, brain pH, and postmortem interval (PMI) for age‐ and sex‐matched cohorts of nine individuals receiving lifetime diagnoses of ASPD and/or CD (specifically, four individuals with ASPD diagnosis only, three individuals with CD diagnosis only, and two individuals with both diagnoses), and nine individuals with no history of psychiatric disease (Table 1). All ASPD/CD subjects were males and had a history of comorbid drug use. The severity of substance abuse (mild, moderate, or severe) was evaluated using the IICD‐10‐CM criteria. Table 2 lists neuropathological findings for all 18 OFC samples. As detailed below, one of the samples (A2; control) was found to be an outlier after tissue quality control. After exclusion of that sample, no significant differences were found between groups with respect to either demographic characteristic (age and ethnicity distribution) and PMI.

**TABLE 1:** Demographic characteristics of subjects.

Group	ID	Age	Ethnicity	Manner of Death	Antisocial behavior‐related diagnoses	Comorbid drug use	Other comorbid psychiatric disorders
Unaffected Controls	A1	48	White	Accidental	/	/	/
	*A2*	*53*	*White*	*Natural*	/	/	/
	A3	40	White	Natural	/	/	/
	A4	23	Black	Accidental	/	/	/
	A5	42	White	Natural	/	/	/
	A6	46	White	Accidental	/	/	/
	A7	45	White	Natural	/	/	/
	A8	47	Black	Natural	/	/	/
	A9	41	White	Natural	/	/	/
ASPD + CD	B1	45	White	Natural	ASPD	Alcohol (S); Opioids (M); Sedatives (M)	/
	B2	37	White	Accidental	CD (childhood‐onset)	Alcohol (M); Cocaine (Mi); Cannabis (Mi)	Dysthymia
	B3	59	White	Accidental	CD (adolescence‐onset)	Alcohol (S); Opioids (Mi)	Intermittent explosive disorder
	B4	49	White	Natural	ASPD	Alcohol (S); Tobacco (S); Sedatives (M); Opioids (M); Amphetamines (Mi)	Unspecified depressive disorder Borderline personality disorder
	B5	44	Black	Accidental	ASPD	Alcohol (S); Cocaine (S); Cannabis (M); Opioids (Mi)	Major depressive disorder, Single episode
	B6	46	Black	Accidental	ASPD	Cannabis (M); Alcohol (Mi); Opioids (Mi); Sedatives (Mi); Cocaine (Mi)	/
	B7	21	White	Suicide	CD (adolescence‐onset); ASPD	Cannabis (Mi)	Major depressive disorder, Single episode; Learning disorders; Borderline personality disorder
	B8	36	Black	Accidental	CD (unspecified onset); ASPD	Tobacco (S); Opioids (S); Cannabis (M); Alcohol (Mi)	Unspecified depressive disorder; Gambling disorder; ADHD; trauma and stress‐related disorder
	B9	56	White	Accidental	CD (unspecified onset)	Opioids (S); Tobacco (S); Alcohol (M); Cocaine (Mi)	Disruptive, impulse‐control disorder

*Note*: Subject A2 was found to be an outlier and excluded from further analyses.

Abbreviations: ASPD, antisocial personality disorder; CD, conduct disorder.

**TABLE 2:** Characteristics of brain samples used in the study.

Group	ID	PMI (h)	RIN	Neuropathological findings
Unaffected Controls	A1	14.2	9.3	No significant pathological abnormalities
	*A2*	*20.1*	*5.2*	*No significant pathological abnormalities*
	A3	31.0	9.1	No significant pathological abnormalities
	A4	18.5	9.3	No significant pathological abnormalities
	A5	14.6	8.3	No significant pathological abnormalities
	A6	12.4	7.4	Small subarachnoid hemorrhage in right cerebrum consistent with history of blunt force trauma. Amyloid angiopathy in frontal lobe and hippocampus only.
	A7	7.1	8.9	No significant pathological abnormalities
	A8	23.4	8.7	No significant pathological abnormalities
	A9	9.9	8.1	No significant pathological abnormalities
ASPD + CD	B1	8.5	7.4	No significant pathological abnormalities
	B2	23.5	7.6	No significant pathological abnormalities
	B3	21.1	7.6	No significant pathological abnormalities
	B4	22.9	7.0	No significant pathological abnormalities
	B5	10.1	8.6	No significant pathological abnormalities
	B6	23.4	9.0	No significant pathological abnormalities
	B7	20.8	7.2	Acute trauma consistent with gunshot wound through the mid‐pons
	B8	13.7	8.6	Mild enlargement of left lateral ventricle
	B9	14.1	6.7	No significant pathological abnormalities

*Note*: Subject A2 was found to be an outlier and excluded from further analyses.

Abbreviations: ASPD: antisocial personality disorder; CD: conduct disorder; PMI: postmortem interval; RIN: RNA integrity number.

### 2.2 RNA extraction and RNA‐seq

RNA was extracted from 30 mg of brain tissue of 18 male individuals (nine individuals receiving diagnoses of ASPD/CD and nine individuals with no history of psychiatric disease) using the RNeasy Lipid Tissue Mini Kit (Qiagen) in an elution volume of 40 μL of RNase‐free water. RNA purity was assessed by the A260/A280 absorbance ratio using a NanoDrop™ spectrophotometer (ThermoFisher Scientific). RNA extracted from OFC samples was sequenced using NovaSeq Reagent Kit v1.5_150 × 150 bp (100 M read‐pairs) Sequencing (Illumina Inc), and sequencing libraries were prepared using Illumina TruSeq Stranded Total RNA Library Prep Ribo‐Zero Gold kit (Illumina).

### 2.3 Data processing

Raw reads were aligned using *STAR v2.7.5b*,^30^ and the count table was generated using the function *featureCounts*, as implemented in the R‐package *subread*.^31^ Read mapping was assessed using *MultiQC v1.12*,^32^ and samples with <60% of uniquely mapped reads were removed before conducting downstream analysis. Raw counts were imported on *DESeq2*.^33^ After removing genes with less than ten total counts across all the samples, data were transformed with the variance stabilizing method^34^ before running principal components analysis (PCA). Outliers were defined as samples with PC1 or PC2 values above plus or minus three standard deviations from the mean in the first round of PCA. The relationship between confounding factors (RIN, PMI, and age) and gene expression was conducted by correlating the top two principal components using Pearson's *r*. Raw counts were normalized using *DESeq2*, and differential expression analysis was performed between ASPD+CD and unaffected controls (CTL), including the covariates sex, RIN, and PMI. P values were adjusted for multiple testing using the False Discovery Rate (FDR) method.^35^ Genes with adjusted *p* < 0.05 were considered statistically significant differentially expressed genes (DEGs). Pathway analysis was conducted on the DEGs using *clusterProfiler* R‐package,^36^ referencing to the Gene Ontology (GO) database, and adjusting the *p*‐values with the FDR method.

A list of cell‐specific markers was generated using a brain single‐nuclei sequencing dataset from the prefrontal cortex (BA10).^37^ This dataset included 24 Alzheimer's Disease (AD) cases and 24 non‐demented controls (ND) from the Religious Orders Study/Memory and Aging Project (ROSMAP) project.^38^ Detailed procedures can be found in ref. [39]. In brief, data preprocessing was performed using R‐Seurat.^40^ Each gene was assigned to a cell class by modeling a linear regression, with expression levels as the dependent variable and cell type as the predictor. The model was adjusted for sex, ethnicity, age, postmortem interval, and disease status (AD or ND). A gene was designated as specific to a cell type when: (1) the adjusted *p*‐values (FDR) were *p* < 0.05, and (2) the regression coefficient of the enriched cell type exhibited a ratio ≥1.76 compared to the second enriched cell type. The cutoff was established by testing coefficient ratios ranging from 1 to 5 until the variation in the number of unclassified genes stabilized below 0.5%. A list of 5399 cell‐specific genes, identified by HGNC symbols, was obtained.

The enrichment test was conducted by hypergeometric statistics using the function *enrichment* from the R‐package *bc3net*.^41^ We conducted coexpression analysis using Multiscale Embedded Gene Coexpression Network Analysis (MEGENA), which has been demonstrated to outperform existing methods such as WGCNA.^42^ The matrix of raw counts was filtered, excluding genes with less than ten total counts, and normalized by means of the *voom* method.^43^ Then, 50% of the genes with lower median absolute deviation (MAD) were excluded. Coexpression network generation was conducted using the *MEGENA* R‐package^42^ according to the following workflow. First, signed pairwise gene correlations were performed using Pearson's method with 1000 permutations, retaining correlation significant at the 5% *FDR* level (function: *calculate. correlation*). Significantly correlated gene pairs (*FDR* <0.05) were ranked and iteratively tested for planarity to grow a Planar Filtered Network using the Planar Maximally Filtered Graph (*PMFG)* technique (function: *calculate.PFN*). Finally, we conducted a multiscale clustering analysis to identify coexpression modules at different network scale topology, as well as their hub genes (function: *do.MEGENA*). Significant coexpression modules (permuted *p* < 0.05) were retained for downstream analysis. We extracted module eigengenes (the first principal component of the genes module) using the function *moduleEigengenes* from the WGCNA R‐package,^44^ and we computed pairwise differential expression by means of the *limma* R‐package,^45^ adjusting for age, PMI, and RIN. Significantly associated modules were annotated for cell‐specific enrichment and GO functional classes using the same workflow described for differentially expressed genes. Relevant coexpression modules were graphically represented using the R‐package *ggnet*.

## 3 RESULTS

### 3.1 Subjects and sample characteristics

All subjects were males, with an average age of 43.2 years (standard deviation: 9.74 years; range: 21–59 years). The average PMI was 17.18 h (standard deviation: 6.5 h; range: 7.1–31 h), and the average RIN was 8.05 (standard deviation: 1.03; range: 5.2–9.3). Sixteen samples (88.9%) had a RIN >7, and seventeen samples (94.4%) had a RIN >6. All samples were sequenced for a total of 504.1 M reads (average: 28.0 M; range: 22.0–34.7 M). The average percentage mapping rate (uniquely mapped reads) was 70.2% (range: 45.3%–78.7%) (Figure S1). Eleven samples out of 18 (61.1%) showed a mapping rate >70.0%, and fifteen samples (83.3%) showed a mapping rate >65%. All the samples but one (A2) had a mapping rate larger than 60%. The A2 sample was a significant outlier, due to its low mapping rate (45.3%) and a RIN <6, and was therefore excluded from further analyses (Figure S2A). After removing that sample, no further outliers were identified (Figure S2B). We assessed the relationship of the gene expression with the confounding factors (PMI, age, and RIN) using the two top principal components (PCs) by Pearson's r. We did not observe significant correlations between the covariates and gene expression (*p*‐values >0.097), with correlation coefficients (|*r*|) ranging from 0.118 to 0.416 (Figures S3–S5). Despite the lack of statistically significant correlations, we chose to include these covariates in our model as they may still influence gene expression. In some instances, we observed moderate correlations (|*r*| > 0.4), suggesting that these factors could have a meaningful impact on expression patterns even though they were not statistically significant at our chosen threshold.

### 3.2 Comparison of ASPD+CD samples versus unaffected controls

**FIGURE 1:**
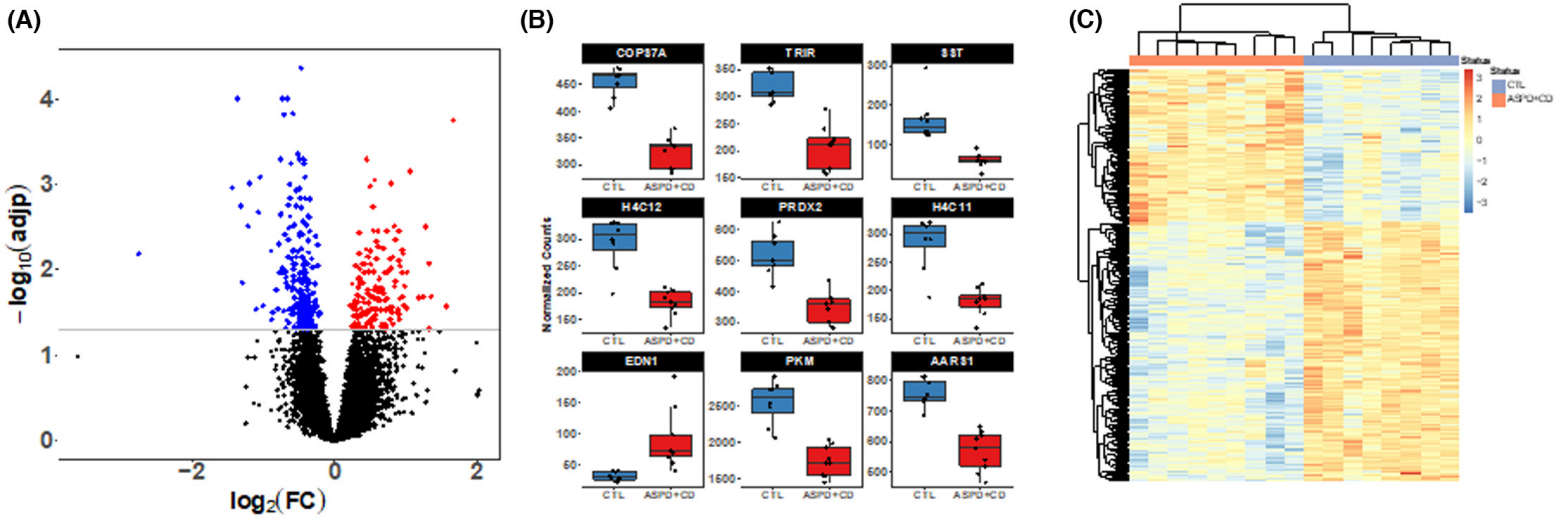
(A). Transcriptomic alterations in the OFC from subjects with ASPD + CD. Log_2_FC plotted relative to −log_10_ *p* value by volcano plot for differentially expressed (DE) transcripts in the OFC. Blue and red squares represent down‐ and upregulated DE transcripts that reach significance, log2FC, and FDR < 0.05 cutoffs, respectively. (B) Differentially expressed genes (DEGs) detected in the comparisons ASPD + CD versus controls. Top DEGs detected in the comparisons ASPD + CD versus CTL. (C) Heatmap showing the DEGs detected in the comparison between ASPD + CD vs CTL clustered by transcript and subject. The heatmap was generated using the Euclidean distance and the Manhattan clustering method. Each column represents a subject (unaffected, blue; ASPD + CD, pink). Subjects within groups cluster together.

The comparison of OFC samples in ASPD+CD subjects vs CTLs showed 328 differentially expressed genes (DEGs) (Table S1 and Figure 1A). Of these, 203 were downregulated, and 125 were upregulated. The boxplots of the top 9 DEGs in ASPD+CD and CTLs are represented in Figure 1B. The heatmap in Figure 1C, generated from the DEGs using the Euclidean distance with the Manhattan clustering method, showed clear‐cut segregation of gene expression differences between ASPD+CD and unaffected controls. Most transcripts were protein‐coding (*n* = 315; 96%). In ASPD+CD samples, astrocyte genes (*n* = 33) were significantly enriched among upregulated genes (FDR = 1.21E‐16), while excitatory neuron genes were significantly enriched among downregulated genes (FDR = 1.84E‐07) (Table S2).

**FIGURE 2:**
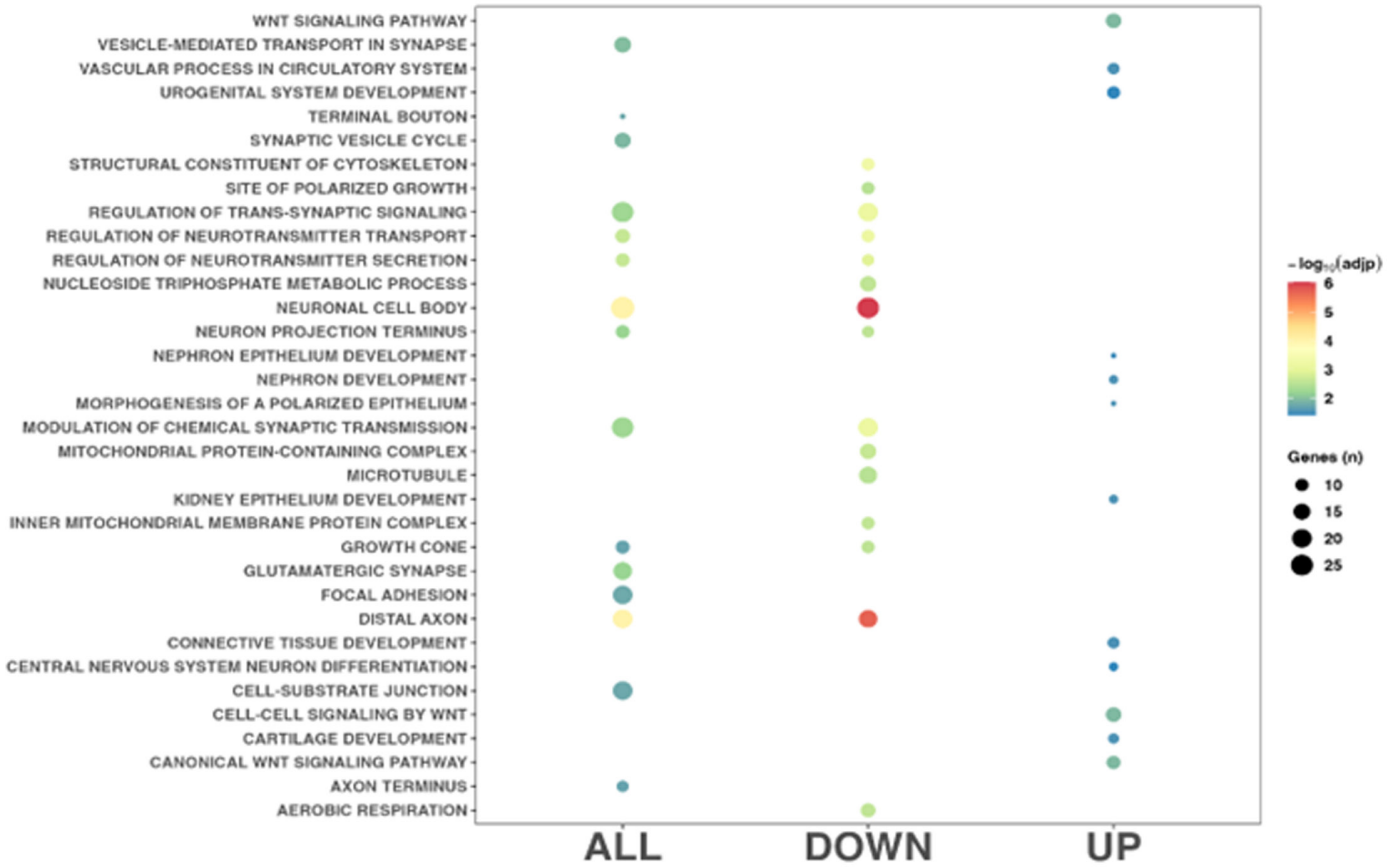
Gene Ontology analysis results conducted using all the differentially expressed genes (all) and downregulated DEGs in ASPD + CD (down), and upregulated genes in ASPD + CD (up). Only the top 15 classes for each group of genes were represented.

As shown in Figure 2 and Table S3, GO analyses revealed significant enrichment for downregulated and upregulated genes (102 and 12 GO classes, respectively). The top downregulated biological process (BP) pathways included “regulation of neurotransmitter transport” (GO:0051588), “modulation of chemical synaptic transmission” (GO:0050804), “regulation of trans‐synaptic signaling” (GO:0099177), “regulation of neurotransmitter secretion” (GO:0046928), “aerobic respiration” (GO:0009060), “nucleoside triphosphate metabolic process” (GO:0009141), “purine ribonucleoside triphosphate metabolic process” (GO: 0009205), and “regulation of synaptic plasticity” (GO:0048167). Downregulated cellular component (CC) pathways included “neuronal cell body” (GO:0043025), “distal axon” (GO:0150034), “mitochondrial protein‐containing complex” (GO:0098798), “microtubule” (GO:0005874), “glutamatergic synapse” (GO:0098978), and “postsynaptic density” (GO:0014069). Conversely, the top upregulated cellular component (CC) pathways were “neuronal cell body” (GO:0043025), “distal axon” (GO:0150034), “mitochondrial protein‐containing complex” (GO:0098798), “inner mitochondrial membrane protein complex” (GO:0098800), “neuron projection terminus” (GO:0044306), “growth cone” (GO:0030426), “site of polarized growth” (GO:0030427), “microtubule” (GO:0005874), “axon terminus” (GO:0043679), “glutamatergic synapse” (GO:0098978), and “synaptic vesicle” (GO:0008021). Finally, the top upregulated BP pathways were “canonical Wnt signaling pathway” (GO:0060070), “Wnt signaling pathway” (GO:0016055), and “cell‐cell signaling by Wnt” (GO:0198738).

**FIGURE 3:**
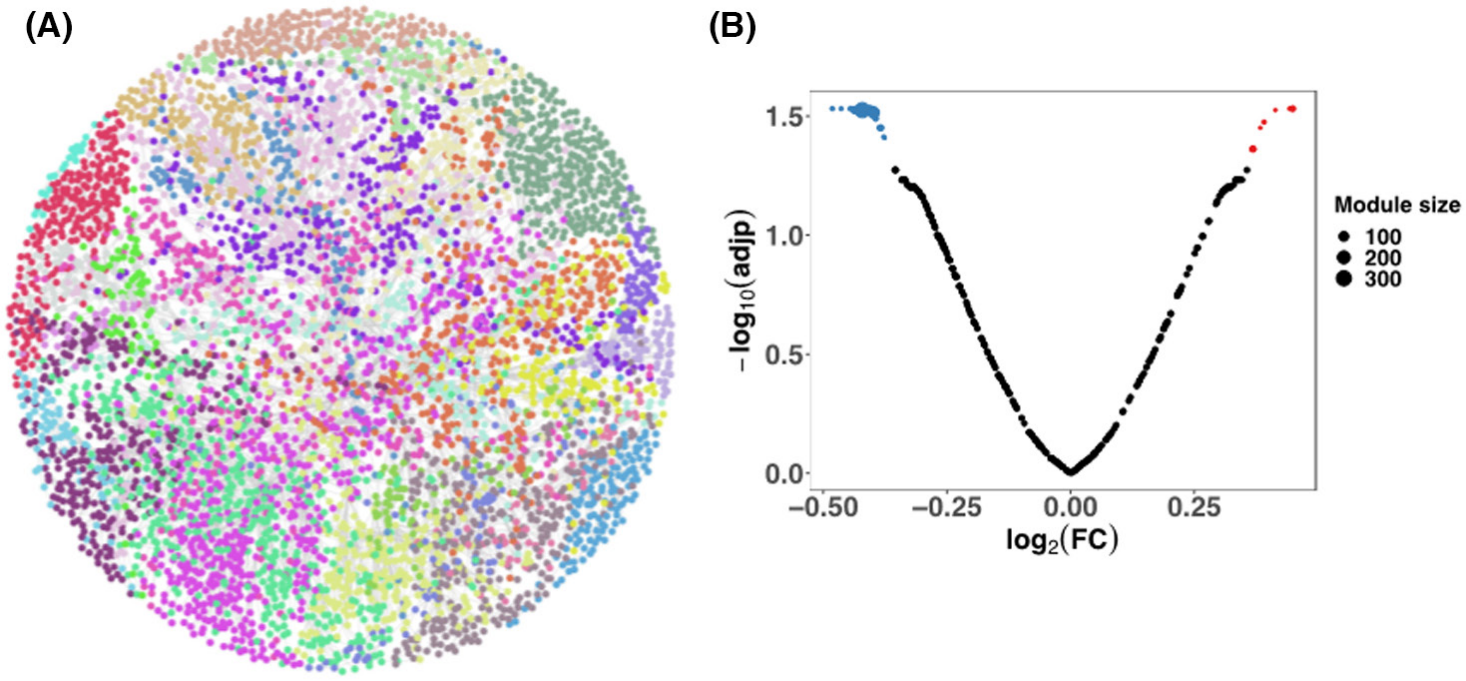
Coexpression network analysis. (A) The coexpression network was generated with the MEGENA method for a total of 308 modules. In the figure, we represented the network indicating the top‐level 29 modules. (B) Volcano plot showing the differential expression of the module eigengenes between ASPD+CD versus CTL. A total of 27 modules were differentially expressed (three of them were top‐level modules: M7, M18, and M2.

**FIGURE 4:**
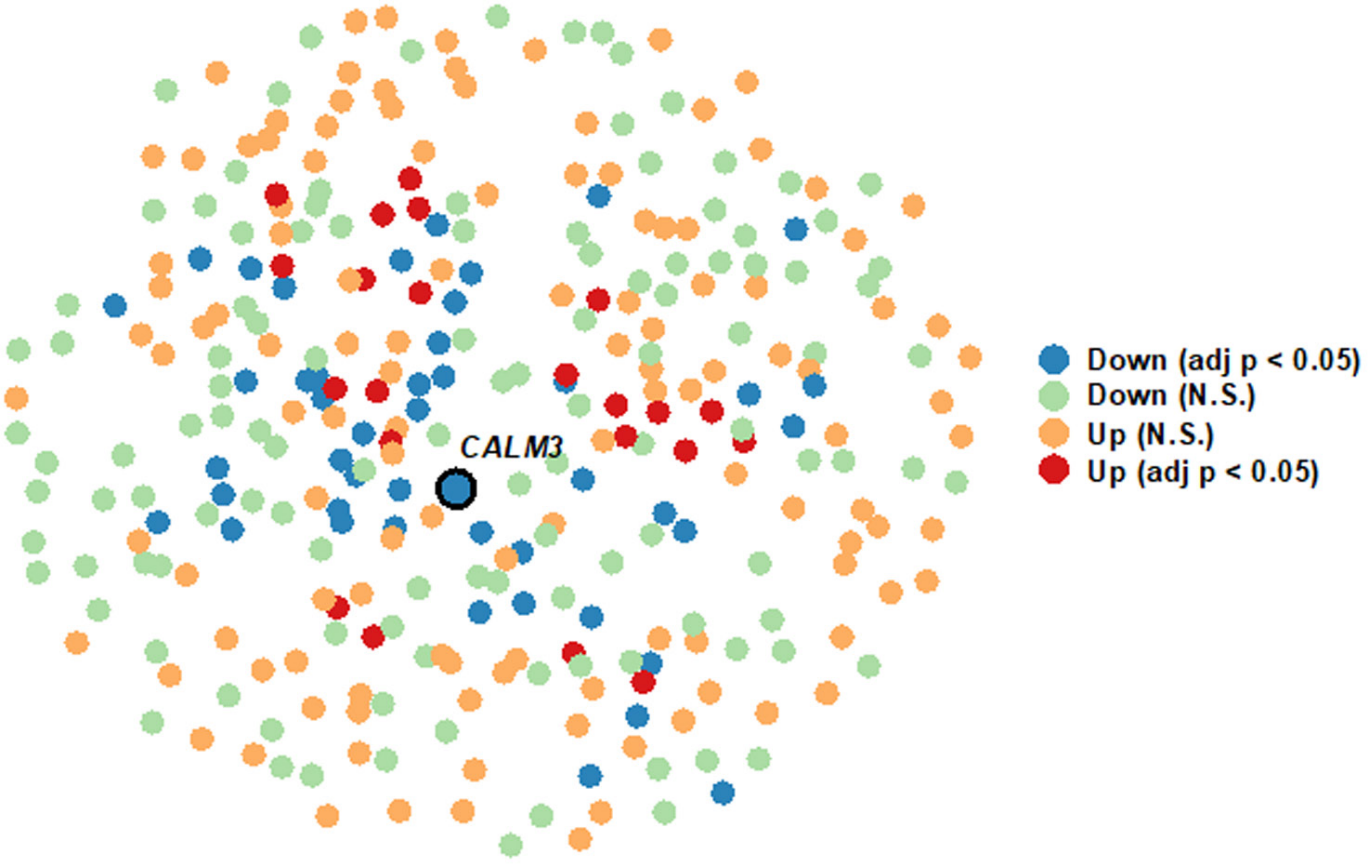
Representation of the most significant functionally relevant coexpression network (M7). The network is downregulated in ASPD+CD versus CTL, enriched for postsynaptic gene ontology (GO) functional classes. Node colors indicate upregulated and downregulated genes statistically significant (adj‐*p* < 0.05) and non‐significant (N.S.) in the differential expression analysis. The label indicates the network hub gene.

We then generated a coexpression network using all 17 samples with 5933 informative genes, including 308 significantly coexpressed modules (Figure 3A). After extracting the module's eigenvalues, we detected 27 modules differentially expressed (19 downregulated and 8 upregulated) between APSD+CD and CTL (Figure 3B). We annotated the significant modules for cell‐specific enrichment and GO functional classes, using the top cells and classes, respectively (Table S4). The complete lists of GO and cell‐type enrichment results are reported in Tables S5 and S6. Among the most functionally relevant coexpression modules, M7 (downregulated) was enriched for the CC “postsynaptic specialization” and the molecular function (MF) “synaptic vesicle recycling” (hub gene: *CALM3*, encoding the protein calmodulin 3) (Figure 4 and Table S5). Another relevant module was M228 (downregulated), enriched for the CC “mitochondrial inner membrane” and the MF “cellular respiration” (hub gene: *EIF6*, encoding the protein Eukaryotic translation initiation factor 6) (Tables S4 and S5).

## 4 DISCUSSION

To the best of our knowledge, this is the first unbiased and comprehensive characterization of gene‐expression alterations in postmortem OFC samples from subjects with ASPD and CD. Consistent with epidemiological evidence showing an extremely high prevalence of SUD in individuals with lifetime diagnoses of ASPD and CD, all subjects in this group had used substances, including alcohol. Additionally, five of the subjects had comorbid mood disorders such as depressive disorders or dysthymia.

The comparison of postmortem OFC samples from individuals with ASPD+CD and age‐matched unaffected controls identified 328 differentially expressed genes (DEGs). Notably, this set did not include key genes associated with antisocial or externalizing behavior (as identified by previous analyses^46^), such as *FOXP2*, *MAOA*, and other serotonin‐related genes. The top downregulated DEG, *COPS7A*, is a component of the COP9 signalosome (CSN), a master regulator of the ubiquitin conjugation pathway.^47^ Several other DEGs implicated in ubiquitination and proteasomal degradation were found to be downregulated in the OFC of subjects with ASPD and/or CD, including *UBQLN4* (ubiquitin 4), *UBA1* (which catalyzes the first step in ubiquitin conjugation), *RAD23A* (which delivers ubiquitinated proteins to the proteasome), and *PSMC3* (a regulator subunit of the 26S proteasome).

The ubiquitin/proteasome pathway allows for the degradation of misfolded or accumulated proteins^48^; importantly, impairments of this machinery have been implicated in the pathophysiology of other developmental problems characterized by social deficits, such as autism‐spectrum disorder.^49^ In line with this concept, the transcript of *COPS7A* was also found to be downregulated in postmortem cortical samples of subjects with autism spectrum disorder and schizophrenia.^50^

Another key DEG that was downregulated in the OFC of ASPD and CD was *SST*, encoding somatostatin. This peptide is mainly produced by one of the main classes of cortical GABAergic interneurons, and its co‐release with GABA has been shown to enhance the inhibitory action of this neurotransmitter.^51^ Interestingly, somatostatin interneurons in the prefrontal cortex have been shown to control affective state discrimination^52^ and social fear.^53^ Therefore, deficits in this molecule may potentially interfere with social information processing, possibly contributing to the ontogeny of antisocial behavior.

GO analyses revealed significant downregulation of several gene clusters related to excitatory neurons of the OFC, namely glutamatergic pyramidal cells. This reduction was observed across key functional domains, namely vesicle and neurotransmitter transport, synaptic organization, ATP biosynthesis, and aerobic respiration. In line with these alterations, most differences were related to neuronal cell body, axon, mitochondria, and glutamatergic synapse/postsynaptic density. Notably, a marked downregulation was found in genes encoding microtubule‐associated molecules, such as tubulin chains (*TUBA1C*, *TUBA4A*, *TUBA8*, *TUBG2*, and *TUBGCP2*) and associated transporters (dynactin subunits *DCTN1* and *DCTN3)*. In line with these findings, the largest module identified by coexpression network analyses (M7) featured the downregulation of transcripts related to synaptic and dendritic modulations. The hub of this module, *CALM3* (also downregulated in OFC samples from ASPD/CD subjects), encodes calmodulin 3, a key regulator of microtubule functions.^54^ Finally, we found the upregulation of two key genes involved in glutamate catabolism: (1) *SLC1A2*, encoding excitatory amino acid transporter 2, the major glutamate transporter in the brain, which is expressed predominantly in astrocytes^55^; and (2) *GLUD1* (glutamate dehydrogenase 1), which catalyzes the degradation of glutamate into α‐ketoglutarate in both astrocytes and neurons, which is also essential to sustain oxidative energy metabolism.^56^ These findings collectively point to a combination of bioenergetic and transport deficits, as well as reductions in postsynaptic density proteins, which likely converge onto alterations of glutamatergic neurotransmission. Such deficits may account for the well‐documented impairments in connectivity between this region and its downstream projections, including the amygdala and other limbic regions.

Finally, our analyses documented the upregulation of Wnt pathways. Wnt proteins are cysteine‐rich glycoproteins associated with different signaling pathways.^57^ Aberrant canonical Wnt/β‐catenin signaling has been shown to contribute to the pathogenesis of several neurological and psychiatric disorders, including autism spectrum disorder, schizophrenia, and mood disorders.^58^ The observed abnormalities in Wnt/β‐catenin signaling may alter OFC architecture, resulting in dysfunctional social processing.

Ample evidence has shown that OFC functional deficits lead to disinhibition, impulsivity, lack of insight, lack of concern with the consequences of one's actions, irresponsibility, social inappropriateness, and deficits in social behavior interpretations.^23^, ^24^, ^25^, ^26^, ^27^ Our results point to the possibility that these impairments may be contributed by specific deficits of the glutamatergic cells and astrocytes, possibly resulting in aberrances of the overall histoarchitectural organization of this brain area. Future proteomic, functional, and histological studies will be necessary to verify whether antisocial behavior is associated with decreased functions or numbers of pyramidal cells in the OFC, and what specific trait components may be associated with specific transcriptomic signatures.

Several limitations of this study should be acknowledged. Firstly, our analyses were conducted on a limited number of brain samples from individuals diagnosed with CD and ASPD. Three of these subjects had a CD, but not ASPD, diagnosis. Notably, their mean age of death was 50.67 years, suggesting that their sociopathic tendencies may have subsided in adulthood, and they had not engaged in pervasive antisocial behavior for more than 30 years. However, the limited power of our analyses does not allow us to verify whether the transcriptomic profile of these individuals may significantly diverge from the others in the same group. Secondly, all subjects were males, reflecting the high sex disproportion observed in ASPD and CD. Thus, it remains unclear whether our results may be generalizable to both genders. Thirdly, all subjects with ASPD and/or CD had a history of drug use, and most had a lifetime diagnosis of mood disorders, reflecting the high comorbidity rates observed in these conditions. Because of this substantial overlap, we cannot exclude that some of the transcriptomic alterations in the OFC may be linked to these comorbid psychiatric entities, rather than ASPD and CD itself. At the same time, it is worth noting that the relationship between ASPD/CD and drug use is complex, and each condition worsens the other in a downward spiral. In addition, these disorders share common vulnerability factors. From this perspective, our data may provide insight into the alterations of the OFC in relation to the comorbidity of ASPD and SUD within the externalizing spectrum.

All these limitations stem primarily from the scarcity of available postmortem brain samples from individuals with diagnoses of ASPD and CD. Although the acquisition of these samples has been made possible by the recent establishment of large brain bank networks, such as the NBB repository in the US, concerted efforts are required to obtain more specimens for targeted analyses on these diagnostic groups. As more postmortem specimens become available, future studies on larger numbers of samples will be needed to corroborate and expand upon the preliminary findings presented in this study.

In conclusion, this study presents the first transcriptomic profiles of the right OFCs in ASPD and CD. Our results suggest that these psychiatric disorders may be characterized by a complex array of functional deficits in the pyramidal neurons of this brain region, including dysfunctional synaptic regulation, microtubule organization, and bioenergetic deficits in pyramidal neurons. The development of novel animal models that may reproduce some of these deficits may prove particularly useful in establishing how these molecular alterations contribute to the ontogeny of antisocial behavior. Such insights could ultimately help establish new leads for the development of more targeted and effective treatments for individuals with ASPD and CD.

## AUTHOR CONTRIBUTIONS

**Ignazio S. Piras**: Bioinformatic analyses, Data Curation, Writing of the first draft of the manuscript. **Giulia Braccagni**: Tissue processing, RNA extraction and sequencing, Data Curation, Editorial Assistance. **Matthew J Huentelman**: Methodology, Data Curation, Editing. **Marco Bortolato**: Conceptualization, Obtainment of tissues, Writing of the manuscript, Editing, Supervision, Supervision, Funding acquisition, Project administration.

## CONFLICT OF INTEREST STATEMENT

The authors declare no conflict of interest.

## Supporting information


Figures S1–S5.

Tables S1–S6.

## Data Availability

The data that support the findings of this study are available from the corresponding author upon reasonable request.
