# Supplementary material for: A preliminary transcriptomic analysis of the orbitofrontal cortex of antisocial individuals
Source: CNS Neurosci Ther. 2023 Jun 2;29(11):3173–82. doi: 10.1111/cns.14283 (PMC10580340; doi:10.1111/cns.14283)

Fig. S1. STAR Mapping rate. All samples but one have a mapping rate (uniquely mapped reads) larger than 60%. We removed sample A2 (CTL) because of the low mapping rate.

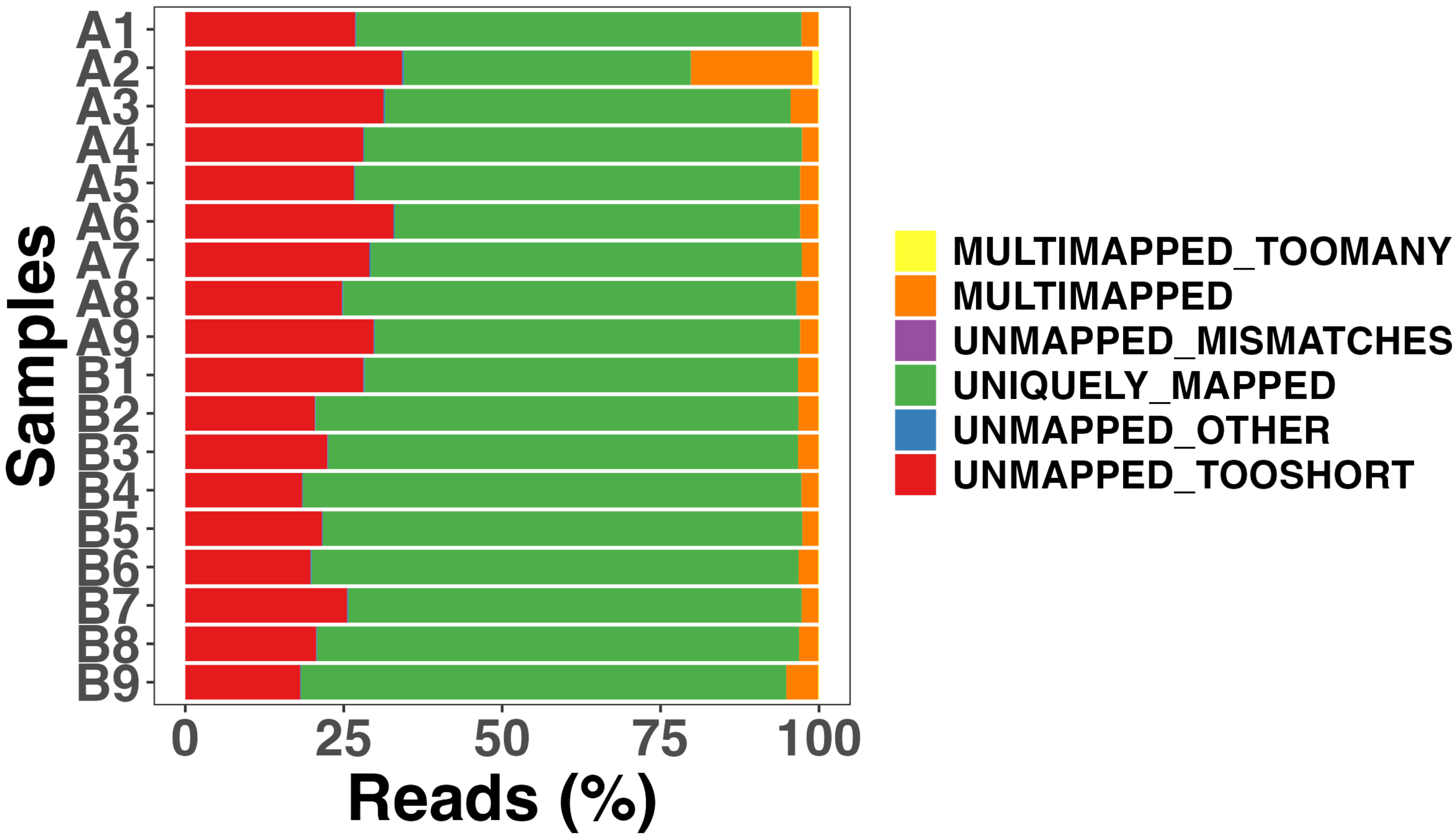

Fig. S2. PCA analysis. The sample A2 had a low mapping rate (< 60%) and was a significant outlier (grey arrow) (A). PCA plot obtained after removing the outlier (B)

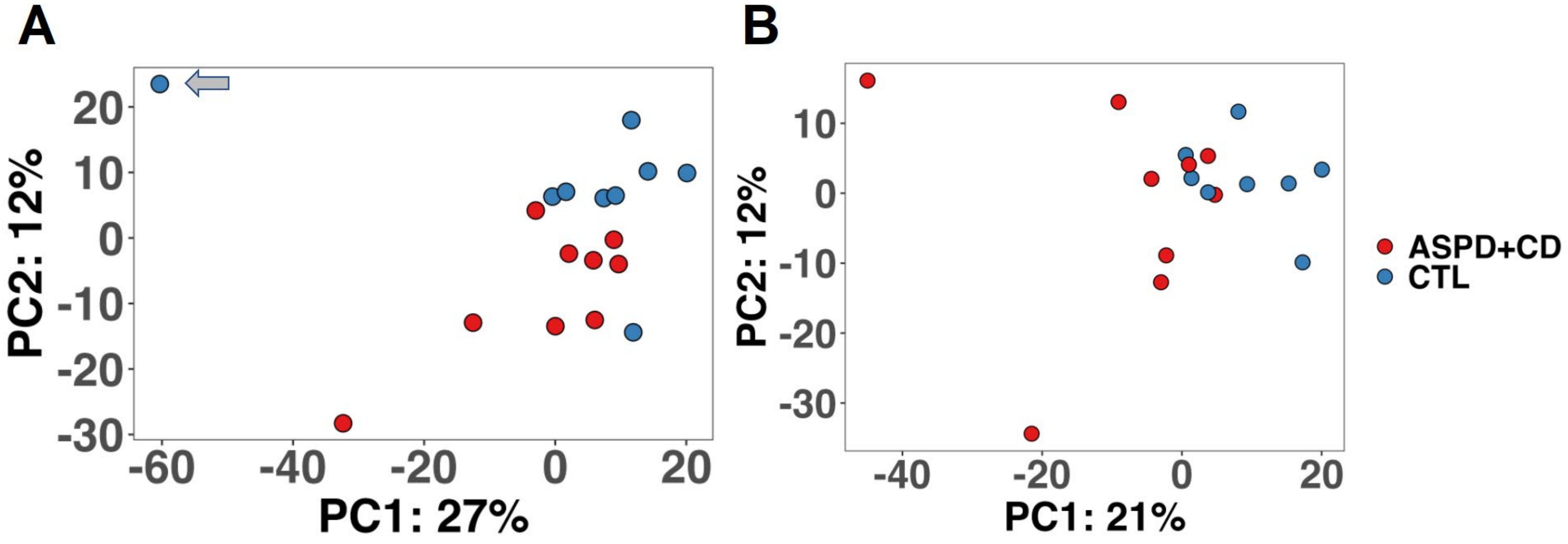

Fig. S3. Correlation between the top two principal components and age.

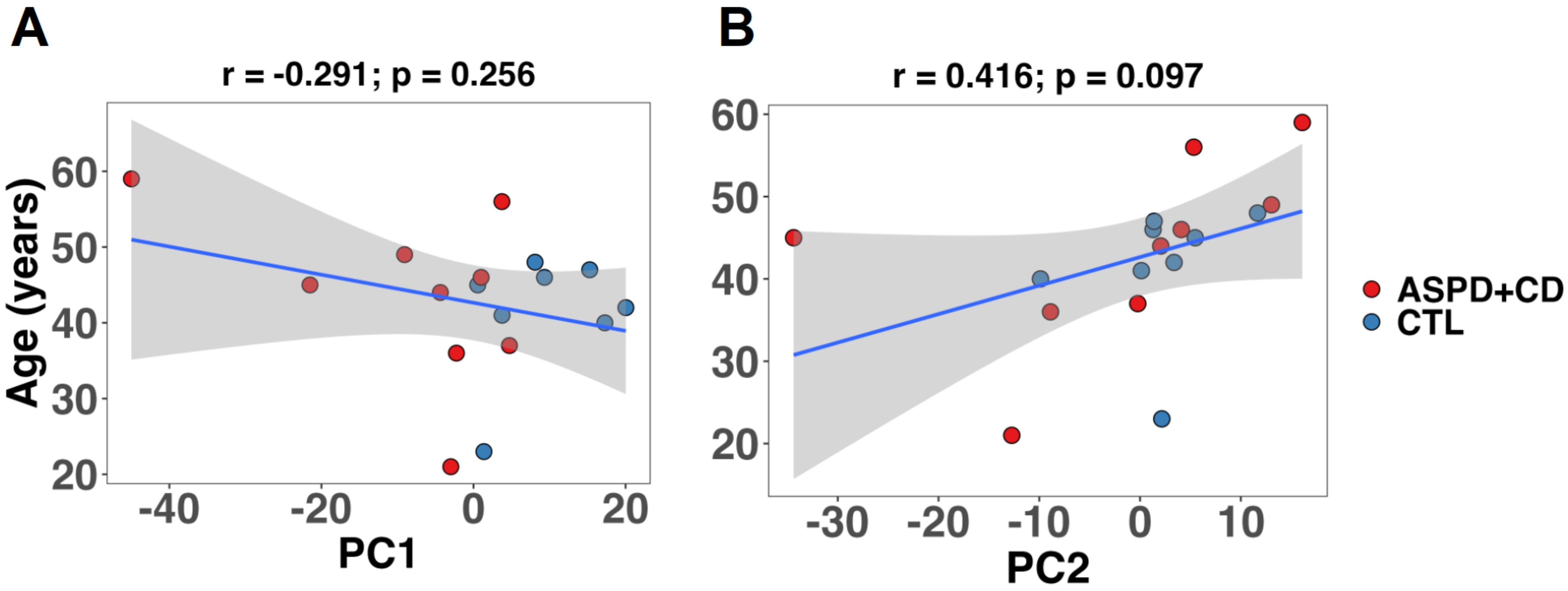

Fig. S4. Correlation between the two top principal components and PMI.

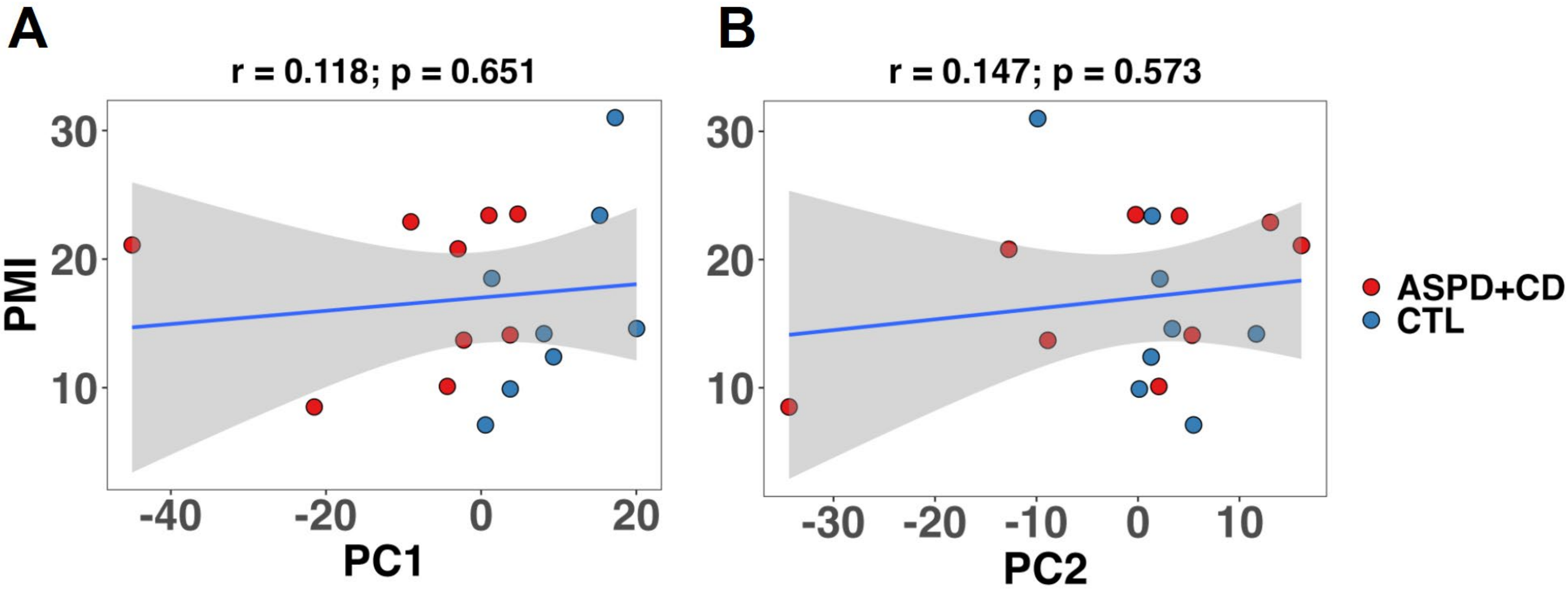

Fig. S5. Correlation between the two top principal components and RIN.

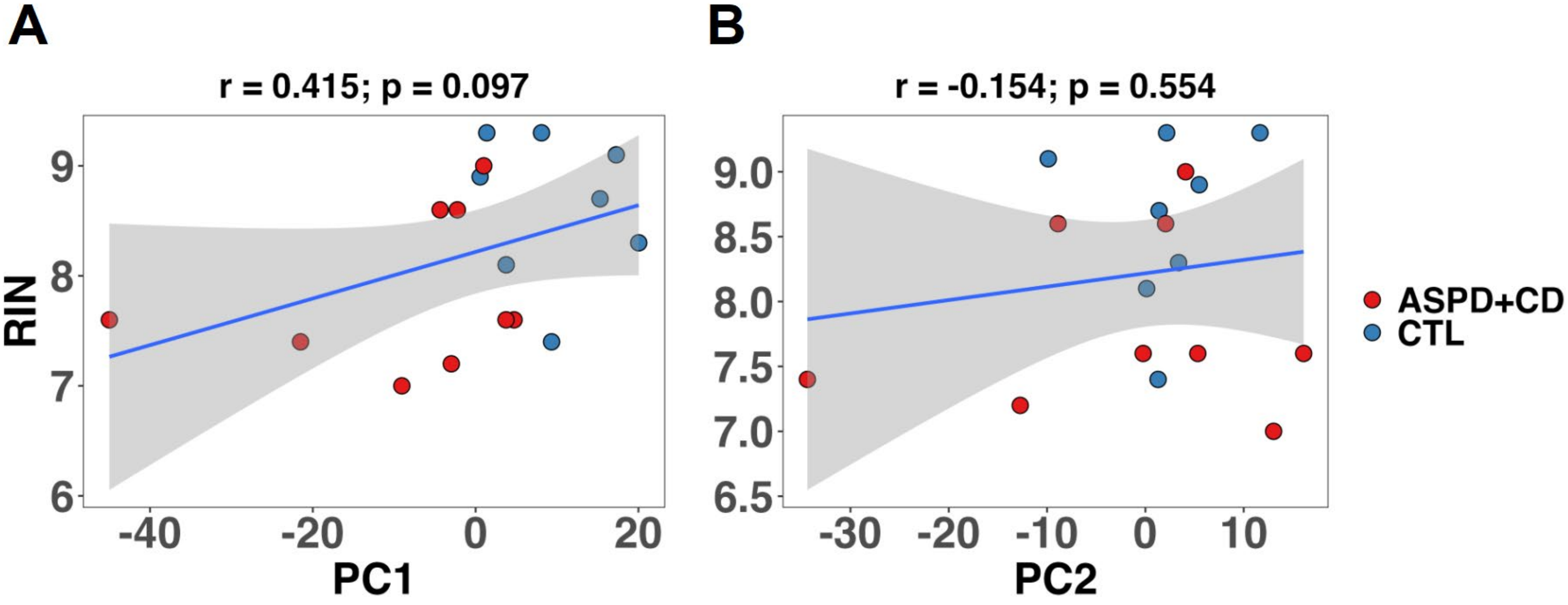

Supplement: Supplementary file 1 — Figures S1–S5. [file CNS-29-3173-s002.pdf]
