# Supplementary material for: A preliminary transcriptomic analysis of the orbitofrontal cortex of antisocial individuals
Source: CNS Neurosci Ther. 2023 Jun 2;29(11):3173–82. doi: 10.1111/cns.14283 (PMC10580340; doi:10.1111/cns.14283)
Supplement: Supplementary file 2 — Tables S1–S6. [file CNS-29-3173-s001.pdf]

Supplementary Table 1: Differentially expressed genes (n=328) in the comparison between ASPD+CD versus CTL. Abbreviations: PC: protein coding; TPP: transcribed processed pseudogene; TUP: transcribed unitary pseudogene; UP: unprocessed pseudogene

Symbol	Ensemble gene ID	Entrez gene ID	baseMean	log2 Fold Change	log2 Fold Change SE	Statistic	p	adj-p	Gene biotype
COPS7A	ENSG00000111652	50813	386.110	-0.479	0.081	-5.935	2.9E-09	4.3E-05	PC
SST	ENSG00000157005	6750	108.993	-1.372	0.247	-5.556	2.8E-08	1.0E-04	PC
TRIR	ENSG00000123144	79002	259.249	-0.660	0.116	-5.670	1.4E-08	1.0E-04	PC
H4C12	ENSG00000273542	8362	234.064	-0.727	0.132	-5.520	3.4E-08	1.0E-04	PC
PRDX2	ENSG00000167815	7001	426.980	-0.585	0.108	-5.418	6.0E-08	1.5E-04	PC
H4C11	ENSG00000197238	8363	228.943	-0.705	0.131	-5.385	7.2E-08	1.5E-04	PC
EDN1	ENSG00000078401	1906	63.761	1.667	0.313	5.332	9.7E-08	1.8E-04	PC
PKM	ENSG00000067225	5315	2128.708	-0.517	0.100	-5.144	2.7E-07	4.4E-04	PC
AARS1	ENSG00000090861	16	656.749	-0.440	0.087	-5.067	4.0E-07	5.1E-04	PC
ATP6V0C	ENSG00000185883	527	598.606	-0.757	0.150	-5.061	4.2E-07	5.1E-04	PC
YPEL3	ENSG00000090238	83719	352.762	-0.494	0.098	-5.065	4.1E-07	5.1E-04	PC
OPHN1	ENSG00000079482	4983	448.794	0.454	0.090	5.029	4.9E-07	5.2E-04	PC
RTL8C	ENSG00000134590	8933	680.874	-0.433	0.086	-5.016	5.3E-07	5.2E-04	PC
KIF5A	ENSG00000155980	3798	4986.696	-0.434	0.087	-4.972	6.6E-07	5.7E-04	PC
SNCB	ENSG00000074317	6620	803.150	-0.615	0.124	-4.974	6.5E-07	5.7E-04	PC
EDNRB	ENSG00000136160	1910	427.607	1.066	0.217	4.922	8.6E-07	7.0E-04	PC
NEFH	ENSG00000100285	4744	893.131	-1.041	0.214	-4.875	1.1E-06	8.2E-04	PC
RAD23A	ENSG00000179262	5886	458.827	-0.412	0.085	-4.871	1.1E-06	8.2E-04	PC
INTU	ENSG00000164066	27152	170.410	0.564	0.116	4.844	1.3E-06	8.9E-04	PC
MIR7-3HG	ENSG00000176840	284424	41.321	-1.199	0.249	-4.806	1.5E-06	9.9E-04	PC
GPAM	ENSG00000119927	57678	191.127	0.794	0.166	4.797	1.6E-06	9.9E-04	lncRNA
NNAT	ENSG00000053438	4826	426.578	-0.630	0.132	-4.785	1.7E-06	1.0E-03	PC
TMEM168	ENSG00000146802	64418	199.119	0.499	0.105	4.766	1.9E-06	1.1E-03	PC
GPR3	ENSG00000181773	2827	21.464	-1.439	0.303	-4.752	2.0E-06	1.1E-03	PC
ARF5	ENSG00000004059	381	300.797	-0.491	0.104	-4.739	2.2E-06	1.1E-03	PC
ATP6V1F	ENSG00000128524	9296	264.126	-0.540	0.114	-4.721	2.3E-06	1.2E-03	PC
CPLX1	ENSG00000168993	10815	468.992	-0.628	0.134	-4.675	2.9E-06	1.4E-03	PC
CDC37	ENSG00000105401	11140	372.174	-0.416	0.089	-4.666	3.1E-06	1.5E-03	PC

HMOX2	ENSG00000103415	3163	257.758	-0.483	0.104	-4.659	3.2E-06	1.5E-03	PC
UBB	ENSG00000170315	7314	3322.463	-0.350	0.075	-4.650	3.3E-06	1.5E-03	PC
ARC	ENSG00000198576	23237	123.942	-1.317	0.286	-4.601	4.2E-06	1.8E-03	PC
HINT1	ENSG00000169567	3094	226.743	-0.543	0.118	-4.600	4.2E-06	1.8E-03	PC
F8A1	ENSG00000288722	8263	113.455	-0.650	0.142	-4.595	4.3E-06	1.8E-03	PC
NADK2	ENSG00000152620	133686	277.347	0.536	0.117	4.581	4.6E-06	1.8E-03	PC
FIBP	ENSG00000172500	9158	200.506	-0.467	0.102	-4.571	4.9E-06	1.9E-03	PC
PCP4	ENSG00000183036	5121	40.279	-1.069	0.236	-4.540	5.6E-06	2.1E-03	PC
TUBA1C	ENSG00000167553	84790	2570.294	-0.754	0.167	-4.528	5.9E-06	2.2E-03	PC
SNCG	ENSG00000173267	6623	298.798	-0.748	0.166	-4.515	6.3E-06	2.3E-03	PC
OTUD5	ENSG00000068308	55593	366.069	-0.373	0.083	-4.503	6.7E-06	2.4E-03	PC
TRAPPC1	ENSG00000170043	58485	122.866	-0.624	0.139	-4.496	6.9E-06	2.4E-03	PC
UBQLN4	ENSG00000160803	56893	386.630	-0.351	0.078	-4.489	7.2E-06	2.4E-03	PC
FLOT1	ENSG00000236271	10211	669.863	-0.444	0.100	-4.457	8.3E-06	2.7E-03	PC
HEXIM1	ENSG00000186834	10614	468.882	-0.395	0.089	-4.429	9.5E-06	3.0E-03	PC
PTMS	ENSG00000159335	5763	396.275	-0.563	0.127	-4.428	9.5E-06	3.0E-03	PC
COX6A1	ENSG00000111775	1337	358.134	-0.580	0.131	-4.418	9.9E-06	3.1E-03	PC
EGR4	ENSG00000135625	1961	39.086	-1.217	0.276	-4.406	1.1E-05	3.1E-03	PC
NKAIN3	ENSG00000185942	286183	140.371	0.838	0.190	4.406	1.1E-05	3.1E-03	PC
ADAMTS9-AS1	ENSG00000241158	101929335	27.238	1.285	0.292	4.399	1.1E-05	3.1E-03	PC
GLRX5	ENSG00000182512	51218	118.761	-0.645	0.147	-4.385	1.2E-05	3.3E-03	lncRNA
RBM5-AS1	ENSG00000281691	100775107	127.315	0.546	0.125	4.369	1.3E-05	3.5E-03	PC
PLSCR4	ENSG00000114698	57088	127.211	0.908	0.208	4.362	1.3E-05	3.5E-03	lncRNA
ZNF883	ENSG00000228623	169834	132.504	0.608	0.140	4.356	1.3E-05	3.6E-03	PC
WBP2	ENSG00000132471	23558	877.520	-0.353	0.081	-4.347	1.4E-05	3.6E-03	PC
LBR	ENSG00000143815	3930	75.766	0.732	0.168	4.350	1.4E-05	3.6E-03	PC
POU2F1	ENSG00000143190	5451	596.759	0.344	0.079	4.334	1.5E-05	3.7E-03	PC
GPR137	ENSG00000173264	56834	174.151	-0.489	0.113	-4.331	1.5E-05	3.7E-03	PC
LIMK1	ENSG00000106683	3984	516.722	-0.445	0.103	-4.314	1.6E-05	3.9E-03	PC
SCRT1	ENSG00000261678	83482	268.083	-0.706	0.164	-4.317	1.6E-05	3.9E-03	PC
BCYRN1	ENSG00000236824	618	4387.528	-0.492	0.114	-4.306	1.7E-05	3.9E-03	PC
ZBTB4	ENSG00000283868	57659	1520.687	-0.271	0.063	-4.296	1.7E-05	4.1E-03	scRNA
FGF2	ENSG00000138685	2247	249.833	0.851	0.199	4.274	1.9E-05	4.4E-03	PC
FARSA	ENSG00000179115	2193	310.948	-0.376	0.088	-4.263	2.0E-05	4.5E-03	PC

PRDX5	ENSG00000126432	25824	129.805	-0.649	0.152	-4.265	2.0E-05	4.5E-03	PC
CFL1	ENSG00000172757	1072	1508.555	-0.370	0.087	-4.254	2.1E-05	4.6E-03	PC
PLEKHG1	ENSG00000120278	57480	209.098	0.590	0.139	4.249	2.1E-05	4.6E-03	PC
NDUFA11	ENSG00000174886	126328	44.532	-0.784	0.186	-4.224	2.4E-05	5.1E-03	PC
PRPF8	ENSG00000174231	10594	2036.889	-0.241	0.057	-4.219	2.5E-05	5.1E-03	PC
ZNF107	ENSG00000196247	51427	134.666	0.611	0.145	4.206	2.6E-05	5.3E-03	PC
NTSR2	ENSG00000169006	23620	60.547	0.682	0.163	4.192	2.8E-05	5.6E-03	PC
CAPNS1	ENSG00000126247	826	552.143	-0.458	0.109	-4.183	2.9E-05	5.7E-03	PC
CDC73	ENSG00000134371	79577	272.740	0.443	0.106	4.180	2.9E-05	5.7E-03	PC
RANBP3L	ENSG00000164188	202151	281.533	0.953	0.229	4.170	3.1E-05	5.9E-03	PC
PIN1	ENSG00000127445	5300	369.093	-0.572	0.137	-4.168	3.1E-05	5.9E-03	PC
MYPOP	ENSG00000176182	339344	97.758	-0.553	0.133	-4.158	3.2E-05	6.0E-03	PC
ATP5MJ	ENSG00000156411	9556	143.020	-0.597	0.144	-4.156	3.2E-05	6.0E-03	PC
KDM1B	ENSG00000165097	221656	106.344	0.508	0.122	4.151	3.3E-05	6.1E-03	PC
VGF	ENSG00000128564	7425	431.322	-0.892	0.215	-4.147	3.4E-05	6.1E-03	PC
FMNL1	ENSG00000184922	752	211.431	-0.507	0.122	-4.148	3.3E-05	6.1E-03	PC
ABCF3	ENSG00000161204	55324	325.636	-0.360	0.087	-4.135	3.6E-05	6.3E-03	PC
XRN1	ENSG00000114127	54464	408.717	0.341	0.083	4.128	3.7E-05	6.4E-03	PC
ACSS3	ENSG00000111058	79611	87.736	0.959	0.233	4.124	3.7E-05	6.4E-03	PC
NPAS4	ENSG00000174576	266743	36.162	-2.758	0.670	-4.116	3.9E-05	6.6E-03	PC
PCSK1N	ENSG00000102109	27344	473.545	-0.458	0.112	-4.094	4.2E-05	7.1E-03	PC
YJEFN3	ENSG00000250067	374887	147.860	-0.538	0.132	-4.089	4.3E-05	7.1E-03	PC
TBC1D3	ENSG00000274611	414060	196.460	0.475	0.116	4.088	4.4E-05	7.1E-03	PC
MYBPC1	ENSG00000196091	4604	35.504	0.820	0.200	4.094	4.2E-05	7.1E-03	PC
NRGN	ENSG00000154146	4900	2564.686	-0.657	0.161	-4.086	4.4E-05	7.1E-03	PC
FSIP2	ENSG00000188738	401024	174.408	0.590	0.145	4.071	4.7E-05	7.5E-03	PC
SYP	ENSG00000102003	6855	3147.030	-0.607	0.149	-4.067	4.8E-05	7.5E-03	PC
USP5	ENSG00000111667	8078	400.637	-0.322	0.079	-4.066	4.8E-05	7.5E-03	PC
GRAMD1C	ENSG00000178075	54762	78.320	0.777	0.191	4.061	4.9E-05	7.6E-03	PC
TBCB	ENSG00000105254	1155	262.750	-0.418	0.103	-4.048	5.2E-05	7.8E-03	PC
SLC1A2	ENSG00000110436	6506	29959.396	0.900	0.222	4.048	5.2E-05	7.8E-03	PC
CKB	ENSG00000166165	1152	2899.910	-0.382	0.095	-4.026	5.7E-05	8.4E-03	PC
HEYL	ENSG00000163909	26508	39.974	1.332	0.331	4.027	5.7E-05	8.4E-03	PC
PTK2B	ENSG00000120899	2185	684.488	-0.371	0.092	-4.019	5.8E-05	8.5E-03	PC

FAN1	ENSG00000276787	22909	280.261	0.389	0.097	4.020	5.8E-05	8.5E-03	PC
POLR2I	ENSG00000105258	5438	101.122	-0.540	0.134	-4.016	5.9E-05	8.6E-03	PC
TMSB4X	ENSG00000205542	7114	987.521	-0.586	0.146	-4.013	6.0E-05	8.6E-03	PC
ZNF680	ENSG00000173041	340252	382.899	0.420	0.105	4.005	6.2E-05	8.6E-03	PC
SMURF2	ENSG00000108854	64750	154.431	0.505	0.126	4.007	6.2E-05	8.6E-03	PC
DDA1	ENSG00000130311	79016	158.564	-0.443	0.111	-4.006	6.2E-05	8.6E-03	PC
GABARAP	ENSG00000170296	11337	295.260	-0.433	0.108	-3.989	6.6E-05	9.0E-03	PC
SMAD4	ENSG00000141646	4089	430.472	0.305	0.076	3.989	6.6E-05	9.0E-03	PC
SPOCK2	ENSG00000107742	9806	2888.607	-0.246	0.062	-3.989	6.6E-05	9.0E-03	PC
SLC25A48	ENSG00000145832	153328	59.440	0.918	0.230	3.985	6.8E-05	9.0E-03	PC
LRPAP1	ENSG00000163956	4043	348.722	-0.313	0.079	-3.972	7.1E-05	9.5E-03	PC
PRRT3	ENSG00000163704	285368	223.264	-0.423	0.107	-3.957	7.6E-05	9.7E-03	PC
GPR153	ENSG00000158292	387509	96.217	-0.583	0.147	-3.958	7.6E-05	9.7E-03	PC
VAMP1	ENSG00000139190	6843	483.988	-0.736	0.186	-3.962	7.4E-05	9.7E-03	PC
ATP13A4	ENSG00000127249	84239	392.082	0.796	0.201	3.960	7.5E-05	9.7E-03	PC
ZNF518A	ENSG00000177853	9849	691.697	0.354	0.089	3.954	7.7E-05	9.8E-03	PC
TMEM35A	ENSG00000126950	59353	272.853	-0.567	0.144	-3.951	7.8E-05	9.8E-03	PC
TBC1D3D	ENSG00000274419	101060389	192.213	0.482	0.123	3.931	8.5E-05	1.0E-02	PC
PGAM4	ENSG00000226784	441531	103.853	-0.688	0.175	-3.933	8.4E-05	1.0E-02	PC
TAS2R14	ENSG00000276541	50840	37.117	0.742	0.189	3.929	8.5E-05	1.0E-02	PC
COX6B1	ENSG00000126267	1340	50.052	-0.730	0.186	-3.921	8.8E-05	1.1E-02	PC
BMPR1B	ENSG00000138696	658	180.107	1.022	0.260	3.924	8.7E-05	1.1E-02	PC
ZNRF3	ENSG00000183579	84133	315.409	0.553	0.141	3.921	8.8E-05	1.1E-02	PC
DRAP1	ENSG00000175550	10589	214.371	-0.447	0.114	-3.905	9.4E-05	1.1E-02	PC
XRCC6	ENSG00000196419	2547	315.582	-0.400	0.102	-3.911	9.2E-05	1.1E-02	PC
CCDC184	ENSG00000177875	387856	227.365	-0.702	0.180	-3.910	9.2E-05	1.1E-02	PC
RAVER2	ENSG00000162437	55225	175.617	0.412	0.106	3.905	9.4E-05	1.1E-02	PC
ANKRD36BP1	ENSG00000214262	84832	54.857	0.946	0.242	3.910	9.2E-05	1.1E-02	PC
CAV2	ENSG00000105971	858	71.214	0.714	0.183	3.904	9.4E-05	1.1E-02	TPP
GNG3	ENSG00000162188	2785	860.270	-0.490	0.126	-3.897	9.7E-05	1.1E-02	PC
PSMC3	ENSG00000165916	5702	205.045	-0.425	0.109	-3.895	9.8E-05	1.1E-02	PC
PDLIM7	ENSG00000196923	9260	42.677	-0.776	0.199	-3.896	9.8E-05	1.1E-02	PC
SPRY4	ENSG00000187678	81848	308.500	-0.572	0.148	-3.874	1.1E-04	1.2E-02	PC
RASGRF1	ENSG00000058335	5923	698.769	-0.419	0.108	-3.871	1.1E-04	1.2E-02	PC

KIF17	ENSG00000117245	57576	85.605	-0.680	0.176	-3.863	1.1E-04	1.2E-02	PC
NKTR	ENSG00000114857	4820	1514.902	0.333	0.087	3.847	1.2E-04	1.3E-02	PC
TUBA4A	ENSG00000127824	7277	1216.398	-0.488	0.127	-3.847	1.2E-04	1.3E-02	PC
AMER2	ENSG00000165566	219287	1873.718	0.357	0.093	3.845	1.2E-04	1.3E-02	PC
ZNF100	ENSG00000274746	163227	116.386	0.625	0.163	3.840	1.2E-04	1.3E-02	PC
GDI1	ENSG00000203879	2664	3164.361	-0.383	0.100	-3.840	1.2E-04	1.3E-02	PC
SBF2	ENSG00000133812	81846	297.034	0.343	0.089	3.837	1.2E-04	1.3E-02	PC
STX1B	ENSG00000099365	112755	1926.069	-0.435	0.114	-3.828	1.3E-04	1.3E-02	PC
EEF1A2	ENSG00000101210	1917	635.151	-0.507	0.132	-3.830	1.3E-04	1.3E-02	PC
CEND1	ENSG00000184524	51286	768.626	-0.428	0.112	-3.827	1.3E-04	1.3E-02	PC
SEMA7A	ENSG00000288455	8482	164.784	-0.465	0.121	-3.826	1.3E-04	1.3E-02	PC
HEPH	ENSG00000089472	9843	60.351	0.860	0.225	3.820	1.3E-04	1.3E-02	PC
ZMAT2	ENSG00000146007	153527	304.234	-0.573	0.150	-3.816	1.4E-04	1.4E-02	PC
CALM3	ENSG00000160014	808	9336.494	-0.442	0.116	-3.814	1.4E-04	1.4E-02	PC
SLC4A4	ENSG00000080493	8671	845.603	0.853	0.224	3.810	1.4E-04	1.4E-02	PC
ASPH	ENSG00000198363	444	583.224	0.366	0.096	3.804	1.4E-04	1.4E-02	PC
EGR2	ENSG00000122877	1959	36.337	-1.300	0.344	-3.782	1.6E-04	1.4E-02	PC
ABHD14A	ENSG00000248487	25864	134.067	-0.458	0.121	-3.789	1.5E-04	1.4E-02	PC
ST8SIA5	ENSG00000101638	29906	315.320	-0.459	0.121	-3.795	1.5E-04	1.4E-02	PC
NFIB	ENSG00000147862	4781	1280.472	0.359	0.094	3.798	1.5E-04	1.4E-02	PC
NME1	ENSG00000239672	4830	126.799	-0.683	0.180	-3.788	1.5E-04	1.4E-02	PC
RPL28	ENSG00000108107	6158	307.966	-0.383	0.101	-3.792	1.5E-04	1.4E-02	PC
SUPT5H	ENSG00000196235	6829	412.826	-0.365	0.096	-3.788	1.5E-04	1.4E-02	PC
YIPF4	ENSG00000119820	84272	196.961	0.390	0.103	3.785	1.5E-04	1.4E-02	PC
TSR2	ENSG00000158526	90121	343.695	-0.361	0.096	-3.782	1.6E-04	1.4E-02	PC
KDM5A	ENSG00000073614	5927	545.338	0.249	0.066	3.774	1.6E-04	1.5E-02	PC
ELMO1	ENSG00000155849	9844	603.286	-0.272	0.072	-3.773	1.6E-04	1.5E-02	PC
ITPRIP	ENSG00000148841	85450	46.919	0.979	0.261	3.757	1.7E-04	1.5E-02	PC
CITED2	ENSG00000164442	10370	232.773	-0.657	0.175	-3.754	1.7E-04	1.6E-02	PC
ADORA2B	ENSG00000170425	136	50.258	0.842	0.225	3.749	1.8E-04	1.6E-02	PC
SDHA	ENSG00000073578	6389	260.085	-0.391	0.104	-3.749	1.8E-04	1.6E-02	PC
NDUFA13	ENSG00000186010	51079	172.475	-0.596	0.159	-3.744	1.8E-04	1.6E-02	PC
UTY	ENSG00000183878	7404	320.250	0.330	0.088	3.742	1.8E-04	1.6E-02	PC
CHGA	ENSG00000100604	1113	827.214	-0.584	0.156	-3.739	1.8E-04	1.6E-02	PC

PRSS23	ENSG00000150687	11098	117.989	0.569	0.152	3.737	1.9E-04	1.6E-02	PC
ITGB8	ENSG00000105855	3696	760.660	0.631	0.169	3.732	1.9E-04	1.6E-02	PC
CLEC2L	ENSG00000236279	154790	69.114	-0.658	0.176	-3.730	1.9E-04	1.6E-02	PC
NPHP1	ENSG00000144061	4867	47.393	0.665	0.179	3.721	2.0E-04	1.7E-02	PC
DCTPP1	ENSG00000179958	79077	32.619	-0.820	0.221	-3.717	2.0E-04	1.7E-02	PC
IDH3B	ENSG00000101365	3420	296.250	-0.382	0.103	-3.711	2.1E-04	1.7E-02	PC
IDH3G	ENSG00000067829	3421	151.963	-0.388	0.105	-3.712	2.1E-04	1.7E-02	PC
HMGN3	ENSG00000118418	9324	135.823	0.466	0.126	3.711	2.1E-04	1.7E-02	PC
CACNA2D2	ENSG00000007402	9254	151.567	-0.477	0.129	-3.705	2.1E-04	1.7E-02	PC
MRPL4	ENSG00000105364	51073	67.305	-0.592	0.160	-3.703	2.1E-04	1.7E-02	PC
COL4A3	ENSG00000169031	1285	65.162	0.564	0.152	3.699	2.2E-04	1.7E-02	PC
RAB3A	ENSG00000105649	5864	466.723	-0.536	0.145	-3.698	2.2E-04	1.7E-02	PC
RNF5	ENSG00000223767	6048	141.145	-0.498	0.135	-3.699	2.2E-04	1.7E-02	PC
ATP1A1	ENSG00000163399	476	1218.087	-0.480	0.130	-3.687	2.3E-04	1.8E-02	PC
LINC00901	ENSG00000242385	100506724	50.100	0.618	0.168	3.684	2.3E-04	1.8E-02	PC
CORO2B	ENSG00000103647	10391	684.426	-0.295	0.080	-3.674	2.4E-04	1.9E-02	lncRNA
EEF1B2	ENSG00000114942	1933	128.980	-0.488	0.133	-3.674	2.4E-04	1.9E-02	PC
LUZP2	ENSG00000187398	338645	208.435	0.468	0.127	3.674	2.4E-04	1.9E-02	PC
POLE2	ENSG00000100479	5427	39.903	0.683	0.186	3.672	2.4E-04	1.9E-02	PC
USP11	ENSG00000102226	8237	1657.719	-0.326	0.089	-3.667	2.5E-04	1.9E-02	PC
ZSWIM6	ENSG00000130449	57688	294.947	0.318	0.087	3.658	2.5E-04	2.0E-02	PC
ADORA1	ENSG00000163485	134	310.772	-0.370	0.101	-3.654	2.6E-04	2.0E-02	PC
SCFD1	ENSG00000092108	23256	93.856	0.501	0.137	3.646	2.7E-04	2.0E-02	PC
GLUD1	ENSG00000148672	2746	1227.886	0.499	0.137	3.645	2.7E-04	2.0E-02	PC
PPP1CA	ENSG00000172531	5499	163.334	-0.430	0.118	-3.646	2.7E-04	2.0E-02	PC
ZNF302	ENSG00000089335	55900	371.497	0.315	0.086	3.647	2.7E-04	2.0E-02	PC
MBOAT2	ENSG00000143797	129642	631.759	0.334	0.092	3.642	2.7E-04	2.0E-02	PC
ANKRD26P3	ENSG00000237636	100101938	20.917	1.362	0.374	3.637	2.8E-04	2.0E-02	PC
CAPZB	ENSG00000077549	832	306.388	-0.423	0.116	-3.638	2.7E-04	2.0E-02	UP
TUBG2	ENSG00000037042	27175	534.806	-0.377	0.104	-3.634	2.8E-04	2.1E-02	PC
SYCP2L	ENSG00000153157	221711	54.008	0.949	0.262	3.629	2.8E-04	2.1E-02	PC
LRAT	ENSG00000121207	9227	19.257	1.245	0.343	3.627	2.9E-04	2.1E-02	PC
USP17L20	ENSG00000250745	100287441	22.862	1.184	0.327	3.619	3.0E-04	2.1E-02	PC
ROGDI	ENSG00000067836	79641	285.422	-0.412	0.114	-3.618	3.0E-04	2.1E-02	PC

TBC1D3I	ENSG00000282144	102724862	149.497	0.549	0.152	3.611	3.0E-04	2.1E-02	PC
FCHO2	ENSG00000157107	115548	164.629	0.397	0.110	3.611	3.0E-04	2.1E-02	PC
S1PR1	ENSG00000170989	1901	334.968	0.616	0.171	3.613	3.0E-04	2.1E-02	PC
TTTY10	ENSG00000229236	246119	34.648	0.741	0.205	3.613	3.0E-04	2.1E-02	PC
TUBA8	ENSG00000183785	51807	168.975	-0.563	0.156	-3.613	3.0E-04	2.1E-02	lncRNA
RHOQ-AS1	ENSG00000250116	100506142	24.814	0.917	0.254	3.605	3.1E-04	2.2E-02	PC
ATP5F1D	ENSG00000099624	513	134.910	-0.474	0.131	-3.606	3.1E-04	2.2E-02	lncRNA
KCND3	ENSG00000171385	3752	608.525	-0.264	0.073	-3.596	3.2E-04	2.2E-02	PC
GATM	ENSG00000171766	2628	399.375	0.607	0.169	3.593	3.3E-04	2.2E-02	PC
GET3	ENSG00000198356	439	132.743	-0.470	0.131	-3.593	3.3E-04	2.2E-02	PC
CORO1A	ENSG00000102879	11151	264.235	-0.424	0.118	-3.591	3.3E-04	2.3E-02	PC
PAIP2	ENSG00000120727	51247	449.687	-0.364	0.101	-3.585	3.4E-04	2.3E-02	PC
NPY6R	ENSG00000226306	4888	53.902	0.706	0.197	3.580	3.4E-04	2.3E-02	PC
ANKRD10	ENSG00000088448	55608	379.377	0.283	0.079	3.580	3.4E-04	2.3E-02	TUP
KLC2	ENSG00000174996	64837	439.723	-0.422	0.118	-3.583	3.4E-04	2.3E-02	PC
STK17B	ENSG00000081320	9262	77.218	0.634	0.177	3.581	3.4E-04	2.3E-02	PC
UBA1	ENSG00000130985	7317	930.054	-0.287	0.081	-3.568	3.6E-04	2.4E-02	PC
GPS2	ENSG00000288325	2874	246.224	-0.370	0.104	-3.564	3.7E-04	2.4E-02	PC
SH3GLB1	ENSG00000097033	51100	452.524	0.317	0.089	3.565	3.6E-04	2.4E-02	PC
ZNF266	ENSG00000174652	10781	157.128	0.483	0.136	3.560	3.7E-04	2.4E-02	PC
TRIT1	ENSG00000043514	54802	117.476	0.414	0.116	3.556	3.8E-04	2.4E-02	PC
CKMT1A	ENSG00000223572	548596	555.138	-0.454	0.128	-3.558	3.7E-04	2.4E-02	PC
VSNL1	ENSG00000163032	7447	4394.019	-0.529	0.149	-3.556	3.8E-04	2.4E-02	PC
TMEM63B	ENSG00000137216	55362	271.426	-0.369	0.104	-3.543	4.0E-04	2.6E-02	PC
ENO2	ENSG00000111674	2026	2845.362	-0.436	0.123	-3.541	4.0E-04	2.6E-02	PC
RGS22	ENSG00000132554	26166	24.831	0.974	0.275	3.538	4.0E-04	2.6E-02	PC
PABPC1L2B	ENSG00000184388	645974	130.863	-0.487	0.138	-3.538	4.0E-04	2.6E-02	PC
COPA	ENSG00000122218	1314	693.330	-0.255	0.072	-3.533	4.1E-04	2.6E-02	PC
UCHL1	ENSG00000154277	7345	1013.967	-0.555	0.157	-3.531	4.1E-04	2.6E-02	PC
TNIK	ENSG00000154310	23043	546.800	0.290	0.082	3.527	4.2E-04	2.6E-02	PC
ACOX1	ENSG00000161533	51	526.305	0.273	0.077	3.528	4.2E-04	2.6E-02	PC
UBR5	ENSG00000104517	51366	850.246	0.217	0.062	3.526	4.2E-04	2.6E-02	PC
DUSP4	ENSG00000120875	1846	103.338	-1.007	0.286	-3.523	4.3E-04	2.6E-02	PC
GNG12	ENSG00000172380	55970	141.366	0.808	0.230	3.518	4.4E-04	2.7E-02	PC

ZNF208	ENSG00000160321	7757	64.937	1.564	0.445	3.516	4.4E-04	2.7E-02	PC
GNAS	ENSG00000087460	2778	4165.019	-0.301	0.086	-3.513	4.4E-04	2.7E-02	PC
EPB41L1	ENSG00000088367	2036	2102.522	-0.244	0.069	-3.510	4.5E-04	2.7E-02	PC
SNX3	ENSG00000112335	8724	632.590	-0.359	0.102	-3.507	4.5E-04	2.7E-02	PC
TTC6	ENSG00000139865	319089	33.217	0.788	0.225	3.505	4.6E-04	2.8E-02	PC
PFN1	ENSG00000108518	5216	94.415	-0.546	0.156	-3.500	4.7E-04	2.8E-02	PC
CAMTA2	ENSG00000108509	23125	713.473	-0.385	0.110	-3.495	4.7E-04	2.8E-02	PC
CRB1	ENSG00000134376	23418	164.145	0.540	0.155	3.494	4.8E-04	2.8E-02	PC
BBS2	ENSG00000125124	583	387.391	0.371	0.106	3.496	4.7E-04	2.8E-02	PC
KLF4	ENSG00000136826	9314	64.164	1.020	0.292	3.495	4.7E-04	2.8E-02	PC
CS	ENSG00000062485	1431	736.649	-0.307	0.088	-3.491	4.8E-04	2.8E-02	PC
CCDC124	ENSG00000007080	115098	85.031	-0.630	0.181	-3.486	4.9E-04	2.9E-02	PC
R3HDM2	ENSG00000179912	22864	539.143	-0.277	0.080	-3.486	4.9E-04	2.9E-02	PC
RPL18	ENSG00000063177	6141	123.474	-0.502	0.144	-3.484	4.9E-04	2.9E-02	PC
FAM241B	ENSG00000171224	219738	69.682	-0.604	0.174	-3.482	5.0E-04	2.9E-02	PC
NDUFB3	ENSG00000119013	4709	43.933	-0.793	0.228	-3.482	5.0E-04	2.9E-02	PC
NPL	ENSG00000135838	80896	64.516	0.723	0.208	3.480	5.0E-04	2.9E-02	PC
TUBGCP2	ENSG00000130640	10844	174.371	-0.412	0.118	-3.477	5.1E-04	2.9E-02	PC
FBXO9	ENSG00000112146	26268	211.028	-0.377	0.109	-3.477	5.1E-04	2.9E-02	PC
TAGLN3	ENSG00000144834	29114	842.933	-0.490	0.141	-3.474	5.1E-04	2.9E-02	PC
ITGAV	ENSG00000138448	3685	564.946	0.511	0.147	3.471	5.2E-04	2.9E-02	PC
KCNN3	ENSG00000143603	3782	594.964	0.573	0.165	3.473	5.1E-04	2.9E-02	PC
PPP1R3C	ENSG00000119938	5507	364.429	0.587	0.169	3.473	5.1E-04	2.9E-02	PC
PANX2	ENSG00000073150	56666	146.322	-0.458	0.132	-3.472	5.2E-04	2.9E-02	PC
LYNX1	ENSG00000180155	66004	1693.935	-0.405	0.117	-3.471	5.2E-04	2.9E-02	PC
DCTN1	ENSG00000204843	1639	2881.224	-0.312	0.090	-3.464	5.3E-04	2.9E-02	PC
RBM12B	ENSG00000183808	389677	437.489	0.314	0.091	3.465	5.3E-04	2.9E-02	PC
LY6E	ENSG00000278032	4061	391.403	-0.473	0.137	-3.465	5.3E-04	2.9E-02	PC
NFIA	ENSG00000162599	4774	895.165	0.530	0.153	3.464	5.3E-04	2.9E-02	PC
TNKS2	ENSG00000107854	80351	454.503	0.270	0.078	3.463	5.3E-04	2.9E-02	PC
DPF1	ENSG00000011332	8193	119.386	-0.480	0.139	-3.464	5.3E-04	2.9E-02	PC
EGR1	ENSG00000120738	1958	379.145	-1.105	0.319	-3.461	5.4E-04	2.9E-02	PC
SHOC1	ENSG00000165181	158401	107.875	0.740	0.214	3.460	5.4E-04	2.9E-02	PC
POLR2F	ENSG00000100142	5435	66.183	-0.646	0.187	-3.459	5.4E-04	2.9E-02	PC

ELOVL2	ENSG00000197977	54898	149.834	0.714	0.207	3.455	5.5E-04	2.9E-02	PC
PPIA	ENSG00000196262	5478	1444.422	-0.400	0.116	-3.450	5.6E-04	2.9E-02	PC
MBNL3	ENSG00000076770	55796	49.948	0.642	0.186	3.451	5.6E-04	2.9E-02	PC
VPS18	ENSG00000104142	57617	258.928	-0.287	0.083	-3.450	5.6E-04	2.9E-02	PC
NAA38	ENSG00000183011	84316	139.504	-0.456	0.132	-3.453	5.5E-04	2.9E-02	PC
CAVIN2	ENSG00000168497	8436	117.497	0.758	0.220	3.451	5.6E-04	2.9E-02	PC
IGSF11-AS1	ENSG00000239877	100506765	22.915	0.893	0.259	3.449	5.6E-04	3.0E-02	PC
APOL2	ENSG00000128335	23780	347.269	-0.478	0.139	-3.440	5.8E-04	3.0E-02	lncRNA
NCKIPSD	ENSG00000213672	51517	429.593	-0.378	0.110	-3.440	5.8E-04	3.0E-02	PC
HAPLN4	ENSG00000187664	404037	426.969	-0.719	0.210	-3.434	6.0E-04	3.1E-02	PC
SMAP2	ENSG00000084070	64744	710.067	-0.382	0.111	-3.434	5.9E-04	3.1E-02	PC
FLOT2	ENSG00000132589	2319	457.049	-0.291	0.085	-3.431	6.0E-04	3.1E-02	PC
THAP2	ENSG00000173451	83591	91.374	0.460	0.134	3.431	6.0E-04	3.1E-02	PC
APBA2	ENSG00000276495	321	1047.981	-0.308	0.090	-3.428	6.1E-04	3.1E-02	PC
P2RY1	ENSG00000169860	5028	79.384	0.884	0.258	3.427	6.1E-04	3.1E-02	PC
HYOU1	ENSG00000149428	10525	1112.182	-0.363	0.106	-3.416	6.4E-04	3.1E-02	PC
NUDC	ENSG00000090273	10726	236.739	-0.396	0.116	-3.421	6.2E-04	3.1E-02	PC
DCTN3	ENSG00000137100	11258	84.870	-0.582	0.170	-3.420	6.3E-04	3.1E-02	PC
PRXL2B	ENSG00000275125	127281	255.170	-0.411	0.120	-3.414	6.4E-04	3.1E-02	PC
PM20D2	ENSG00000146281	135293	118.885	0.388	0.114	3.417	6.3E-04	3.1E-02	PC
CPNE9	ENSG00000144550	151835	77.896	-0.594	0.174	-3.413	6.4E-04	3.1E-02	PC
PLAC4	ENSG00000280109	191585	66.217	0.807	0.237	3.414	6.4E-04	3.1E-02	PC
CACYBP	ENSG00000116161	27101	405.188	-0.331	0.097	-3.413	6.4E-04	3.1E-02	lncRNA
GPATCH4	ENSG00000160818	54865	196.196	-0.387	0.113	-3.419	6.3E-04	3.1E-02	PC
S100PBP	ENSG00000116497	64766	192.180	0.463	0.136	3.412	6.4E-04	3.1E-02	PC
SPTAN1	ENSG00000197694	6709	5936.563	-0.220	0.064	-3.416	6.4E-04	3.1E-02	PC
DYNLRB1	ENSG00000125971	83658	210.712	-0.430	0.126	-3.422	6.2E-04	3.1E-02	PC
MYOZ3	ENSG00000164591	91977	30.216	-0.868	0.254	-3.410	6.5E-04	3.2E-02	PC
MDH2	ENSG00000146701	4191	229.993	-0.386	0.113	-3.408	6.6E-04	3.2E-02	PC
RTL5	ENSG00000242732	340526	299.344	-0.395	0.116	-3.406	6.6E-04	3.2E-02	PC
HUWE1	ENSG00000086758	10075	1944.890	-0.204	0.060	-3.400	6.7E-04	3.2E-02	PC
VDAC2	ENSG00000165637	7417	237.342	-0.404	0.119	-3.400	6.8E-04	3.2E-02	PC
FNDC4	ENSG00000115226	64838	255.024	-0.469	0.138	-3.398	6.8E-04	3.3E-02	PC
NEFM	ENSG00000104722	4741	2518.900	-0.557	0.164	-3.396	6.8E-04	3.3E-02	PC

SLCO1C1	ENSG00000139155	53919	114.448	0.909	0.268	3.389	7.0E-04	3.3E-02	PC
ZNF395	ENSG00000186918	55893	204.508	0.450	0.133	3.390	7.0E-04	3.3E-02	PC
TMEM120A	ENSG00000189077	83862	45.424	-0.598	0.176	-3.390	7.0E-04	3.3E-02	PC
ZNF594	ENSG00000180626	84622	281.968	0.364	0.107	3.386	7.1E-04	3.3E-02	PC
RNF152	ENSG00000176641	220441	269.191	0.379	0.112	3.384	7.1E-04	3.3E-02	PC
WASH2P	ENSG00000291134	375260	182.789	-0.510	0.151	-3.383	7.2E-04	3.3E-02	PC
TAOK2	ENSG00000149930	9344	611.518	-0.224	0.066	-3.383	7.2E-04	3.3E-02	lncRNA
CCT7	ENSG00000135624	10574	158.934	-0.390	0.116	-3.375	7.4E-04	3.4E-02	PC
AHSA1	ENSG00000100591	10598	157.528	-0.449	0.133	-3.375	7.4E-04	3.4E-02	PC
SOCS4	ENSG00000180008	122809	352.123	0.301	0.089	3.373	7.4E-04	3.4E-02	PC
FKBP4	ENSG00000004478	2288	362.365	-0.349	0.103	-3.374	7.4E-04	3.4E-02	PC
LIFR	ENSG00000113594	3977	1047.079	0.581	0.172	3.378	7.3E-04	3.4E-02	PC
MYG1	ENSG00000139637	60314	115.475	-0.454	0.135	-3.373	7.4E-04	3.4E-02	PC
EDF1	ENSG00000107223	8721	307.470	-0.316	0.094	-3.376	7.4E-04	3.4E-02	PC
IMP4	ENSG00000136718	92856	167.827	-0.357	0.106	-3.373	7.4E-04	3.4E-02	PC
ZBTB41	ENSG00000177888	360023	660.090	0.270	0.080	3.370	7.5E-04	3.4E-02	PC
TRPV3	ENSG00000167723	162514	76.132	0.547	0.163	3.362	7.7E-04	3.5E-02	PC
CACNB1	ENSG00000067191	782	524.374	-0.419	0.125	-3.359	7.8E-04	3.5E-02	PC
ASB13	ENSG00000196372	79754	109.048	-0.443	0.132	-3.354	8.0E-04	3.6E-02	PC
DOCK7	ENSG00000116641	85440	247.918	0.324	0.097	3.350	8.1E-04	3.6E-02	PC
ATP6V1E1	ENSG00000131100	529	413.521	-0.498	0.149	-3.337	8.5E-04	3.8E-02	PC
KCNA1	ENSG00000111262	3736	773.248	-0.478	0.143	-3.335	8.5E-04	3.8E-02	PC
SSPN	ENSG00000123096	8082	141.317	0.910	0.273	3.335	8.5E-04	3.8E-02	PC
SLC15A2	ENSG00000163406	6565	250.585	0.569	0.171	3.334	8.6E-04	3.8E-02	PC
CRELD1	ENSG00000163703	78987	313.999	-0.348	0.104	-3.332	8.6E-04	3.8E-02	PC
RAB31	ENSG00000168461	11031	517.712	0.511	0.153	3.330	8.7E-04	3.8E-02	PC
PAX7	ENSG00000009709	5081	42.968	-0.884	0.265	-3.330	8.7E-04	3.8E-02	PC
RPL13AP17	ENSG00000231322	399670	41.323	0.784	0.236	3.327	8.8E-04	3.8E-02	PC

Supplementary Table 2. Cell-specific enrichment for the comparison between OFC samples from subjects with antisocial personality disorder (ASPD) and/or conduct disorder (CD) in comparison with unaffected controls. Astrocyte genes were significantly enriched among upregulated genes in ASPD+CD, whereas Excitatory Neurons genes were significantly enriched among downregulated genes in ASPD +CD (in bold). DEG: differentially expressed genes.

Genes	TermID	Genes	All	p	FDR
**All DEGs**	**Ast**	**36**	**498**	**3.18E-07**	**2.54E-06**
	**Ex**	**56**	**1312**	**1.53E-03**	**6.10E-03**
	In	5	179	5.85E-01	9.99E-01
	Per	3	135	7.47E-01	9.99E-01
	End	4	214	8.66E-01	9.99E-01
	Mic	5	336	9.66E-01	9.99E-01
	Opc	3	274	9.86E-01	9.99E-01
	Oli	2	331	9.99E-01	9.99E-01
**Upregulated**	**Ast**	**33**	**498**	**1.51E-17**	**1.21E-16**
	Per	2	135	4.23E-01	1.00E+00
	End	2	214	6.68E-01	1.00E+00
	Opc	2	274	7.93E-01	1.00E+00
	In	1	179	8.55E-01	1.00E+00
	Oli	1	331	9.72E-01	1.00E+00
	Ex	4	1312	1.00E+00	1.00E+00
	Mic	0	336	1.00E+00	1.00E+00
**Downregulated**	**Ex**	**52**	**1312**	**2.30E-08**	**1.84E-07**
	In	4	179	3.94E-01	9.98E-01
	Mic	5	336	7.17E-01	9.98E-01
	End	2	214	8.97E-01	9.98E-01
	Per	1	135	9.12E-01	9.98E-01
	Opc	1	274	9.93E-01	9.98E-01
	Ast	3	498	9.94E-01	9.98E-01
	Oli	1	331	9.98E-01	9.98E-01

Supplementary Table 3. Gene ontology (GO) functional classes significantly enriched among the differentially expressed genes detected in the comparison between antisocial personality disorder (ASPD) + conduct disorder (CD) vs unaffected controls. DEG: differentially expressed genes.

Genes	Ontology	ID	Description	Gene Ratio	BgRatio	p-value	Adj. p	Count	Gene ID
All DEGs	BP	GO:0051588	regulation of neurotransmitter transport	12/359	105/18903	7.33E-07	2.33E-03	12	CPLX1/SNCG/FLOT1/SYP/STX1B/CALM3/ADORA2B/RAB3A/VPS18/APBA2/P2RY1/CPLX2
	BP	GO:0046928	regulation of neurotransmitter secretion	11/359	91/18903	1.21E-06	2.33E-03	11	CPLX1/SNCG/SYP/STX1B/CALM3/ADORA2B/RAB3A/VPS18/APBA2/P2RY1/CPLX2
	BP	GO:0050804	modulation of chemical synaptic transmission	24/359	448/18903	5.51E-06	5.50E-03	24	EDN1/OPHN1/CPLX1/ARC/SNCG/FLOT1/VGF/NPAS4/NRGN/SYP/PTK2B/RASGRF1/STX1B/CALM3/EGR2/ADORA2B/RAB3A/ADORA1/PAIP2/VPS18/APBA2/P2RY1/ARRB2/CPLX2
	BP	GO:0099177	regulation of trans-synaptic signaling	24/359	449/18903	5.72E-06	5.50E-03	24	EDN1/OPHN1/CPLX1/ARC/SNCG/FLOT1/VGF/NPAS4/NRGN/SYP/PTK2B/RASGRF1/STX1B/CALM3/EGR2/ADORA2B/RAB3A/ADORA1/PAIP2/VPS18/APBA2/P2RY1/ARRB2/CPLX2
	BP	GO:0099003	vesicle-mediated transport in synapse	15/359	212/18903	1.38E-05	1.06E-02	15	OPHN1/SNCB/CPLX1/ARC/SNCG/SYP/STX1B/CALM3/ADORA2B/RAB3A/FCHO2/VPS18/APBA2/P2RY1/CPLX2
	BP	GO:0099504	synaptic vesicle cycle	14/359	192/18903	1.91E-05	1.22E-02	14	OPHN1/SNCB/CPLX1/SNCG/SYP/STX1B/CALM3/ADORA2B/RAB3A/FCHO2/VPS18/APBA2/P2RY1/CPLX2
	BP	GO:1902915	negative regulation of protein polyubiquitination	4/359	11/18903	3.80E-05	2.09E-02	4	GPS2/UBR5/PPIA/OTUB1
	BP	GO:0016079	synaptic vesicle exocytosis	10/359	111/18903	5.07E-05	2.43E-02	10	CPLX1/SYP/STX1B/CALM3/ADORA2B/RAB3A/VPS18/APBA2/P2RY1/CPLX2
	BP	GO:0006836	neurotransmitter transport	14/359	212/18903	5.70E-05	2.43E-02	14	CPLX1/SNCG/FLOT1/SYP/SLC1A2/STX1B/CALM3/ADORA2B/RAB3A/VPS18/APBA2/P2RY1/GABRA2/CPLX2
	BP	GO:0001505	regulation of neurotransmitter levels	14/359	219/18903	8.08E-05	3.11E-02	14	CPLX1/SNCG/FLOT1/SYP/SLC1A2/STX1B/CALM3/ADORA2B/RAB3A/VPS18/APBA2/P2RY1/GABRA2/CPLX2

	BP	GO:0072350	tricarboxylic acid metabolic process	4/359	14/18903	1.10E-04	3.83E-02	4	IDH3B/IDH3G/GLUD1/CS
	BP	GO:0036465	synaptic vesicle recycling	8/359	79/18903	1.26E-04	3.83E-02	8	OPHN1/SNCB/SNCG/SYP/STX1B/CALM3/RAB3A/FCHO2
	BP	GO:2000300	regulation of synaptic vesicle exocytosis	7/359	60/18903	1.37E-04	3.83E-02	7	SYP/CALM3/ADORA2B/RAB3A/VPS18/APBA2/P2RY1
	BP	GO:0007269	neurotransmitter secretion	11/359	151/18903	1.50E-04	3.83E-02	11	CPLX1/SNCG/SYP/STX1B/CALM3/ADORA2B/RAB3A/VPS18/APBA2/P2RY1/CPLX2
	BP	GO:0099643	signal release from synapse	11/359	151/18903	1.50E-04	3.83E-02	11	CPLX1/SNCG/SYP/STX1B/CALM3/ADORA2B/RAB3A/VPS18/APBA2/P2RY1/CPLX2
	BP	GO:0009141	nucleoside triphosphate metabolic process	15/359	267/18903	1.91E-04	4.60E-02	15	PKM/ATP6V0C/NDUFA11/ATP5MJ/TMSB4X/PGAM4/SLC4A4/NME1/SDHA/NDUFA13/DCTPP1/ATP5F1D/ENO2/NDUFB3/ATP5IF1
	BP	GO:0009205	purine ribonucleoside triphosphate metabolic process	14/359	242/18903	2.31E-04	4.75E-02	14	PKM/ATP6V0C/NDUFA11/ATP5MJ/TMSB4X/PGAM4/SLC4A4/NME1/SDHA/NDUFA13/ATP5F1D/ENO2/NDUFB3/ATP5IF1
	BP	GO:1902914	regulation of protein polyubiquitination	5/359	30/18903	2.32E-04	4.75E-02	5	GABARAP/GPS2/UBR5/PPIA/OTUB1
	BP	GO:0050808	synapse organization	20/359	432/18903	2.43E-04	4.75E-02	20	OPHN1/SNCB/NEFH/ARC/SNCG/CFL1/PIN1/NPAS4/SPOCK2/RAB3A/PFN1/DCTN1/NFIA/HAPLN4/CACNB1/SEZ6L2/GABRA2/NEFL/NRG1/CHCHD10
	BP	GO:0006099	tricarboxylic acid cycle	5/359	31/18903	2.72E-04	4.75E-02	5	SDHA/IDH3B/IDH3G/CS/MDH2
	BP	GO:0046034	ATP metabolic process	13/359	217/18903	2.73E-04	4.75E-02	13	PKM/ATP6V0C/NDUFA11/ATP5MJ/TMSB4X/PGAM4/SLC4A4/SDHA/NDUFA13/ATP5F1D/ENO2/NDUFB3/ATP5IF1
	BP	GO:0023061	signal release	21/359	470/18903	2.76E-04	4.75E-02	21	EDN1/EDNRB/NNAT/CPLX1/SNCG/VGF/SYP/SMAD4/STX1B/CALM3/ADORA2B/CHGA/HMGN3/RAB3A/ADORA1/GLUD1/VSNL1/VPS18/APBA2/P2RY1/CPLX2

	BP	GO:0009144	purine nucleoside triphosphate metabolic process	14/359	247/18903	2.84E-04	4.75E-02	14	PKM/ATP6V0C/NDUFA11/ATP5MJ/TMSB4X/PGAM4/SLC4A4/NME1/SDHA/NDUFA13/ATP5F1D/ENO2/NDUFB3/ATP5IF1
	BP	GO:0009199	ribonucleoside triphosphate metabolic process	14/359	249/18903	3.09E-04	4.94E-02	14	PKM/ATP6V0C/NDUFA11/ATP5MJ/TMSB4X/PGAM4/SLC4A4/NME1/SDHA/NDUFA13/ATP5F1D/ENO2/NDUFB3/ATP5IF1
	CC	GO:0043025	neuronal cell body	28/376	497/19869	3.13E-07	9.46E-05	28	SST/KIF5A/SNCB/CPLX1/UBB/ARC/SNCG/GLRX5/VGF/XRN1/NRGN/CKB/PTK2B/GDI1/ADORA1/PPP1CA/ROGDI/KCND3/ENO2/UCHL1/KCNN3/DCTN1/FKBP4/KCNA1/SEZ6L2/GABRA2/FXR2/CPLX2
	CC	GO:0150034	distal axon	20/376	278/19869	3.81E-07	9.46E-05	20	OPHN1/SNCB/CPLX1/SNCG/CFL1/SYP/PTK2B/RASGRF1/CALM3/RAB3A/ADORA1/ENO2/COPA/TAOK2/FKBP4/DOCK7/KCNA1/NEFL/FXR2/CPLX2
	CC	GO:0044306	neuron projection terminus	11/376	131/19869	4.02E-05	6.20E-03	11	OPHN1/SNCB/CPLX1/SNCG/SYP/SLC1A2/RAB3A/ADORA1/UCHL1/KCNA1/CPLX2
	CC	GO:0098978	glutamatergic synapse	18/376	324/19869	4.99E-05	6.20E-03	18	OPHN1/CPLX1/ARC/FLOT1/PIN1/NRGN/SLC1A2/PTK2B/ADORA2B/PPP1CA/CORO1A/TNIK/PFN1/VPS18/FLOT2/P2RY1/KCNA1/NRG1
	CC	GO:0005925	focal adhesion	20/376	422/19869	1.72E-04	1.71E-02	20	ATP6V0C/FLOT1/LIMK1/CFL1/PTK2B/CAV2/PDLIM7/SPRY4/ITGB8/CORO2B/PFN1/RPL18/ITGAV/PPIA/FLOT2/HYOU1/DOCK7/RPS15/CPNE3/SDC4
	CC	GO:0030055	cell-substrate junction	20/376	432/19869	2.34E-04	1.75E-02	20	ATP6V0C/FLOT1/LIMK1/CFL1/PTK2B/CAV2/PDLIM7/SPRY4/ITGB8/CORO2B/PFN1/RPL18/ITGAV/PPIA/FLOT2/HYOU1/DOCK7/RPS15/CPNE3/SDC4
	CC	GO:0043195	terminal bouton	6/376	47/19869	2.46E-04	1.75E-02	6	OPHN1/CPLX1/SYP/RAB3A/ADORA1/CPLX2
	CC	GO:0043679	axon terminus	9/376	115/19869	3.41E-04	1.93E-02	9	OPHN1/SNCB/CPLX1/SNCG/SYP/RAB3A/ADORA1/KCNA1/CPLX2
	CC	GO:0030426	growth cone	11/376	167/19869	3.50E-04	1.93E-02	11	CFL1/PTK2B/RASGRF1/CALM3/ENO2/COPA/TAOK2/FKBP4/DOCK7/NEFL/FXR2
	CC	GO:0098685	Schaffer collateral - CA1 synapse	7/376	72/19869	4.21E-04	2.00E-02	7	NEFH/CPLX1/SYP/ADORA2B/CAPZB/APBA2/NEFL

	CC	GO:0030427	site of polarized growth	11/376	173/19869	4.72E-04	2.00E-02	11	CFL1/PTK2B/RASGRF1/CALM3/ENO2/COPA/TAOK2/FKBP4/DOCK7/NEFL/FXR2
	CC	GO:0030133	transport vesicle	19/376	423/19869	4.84E-04	2.00E-02	19	EDN1/ATP6V0C/TMEM168/ATP6V1F/VGF/NRGN/SYP/VAMP1/CAV2/STX1B/CALM3/CHGA/RAB3A/ROGDI/COPA/APBA2/ATP6V1E1/SSPN/GABRA2
	CC	GO:0098800	inner mitochondrial membrane protein complex	10/376	158/19869	8.76E-04	3.35E-02	10	COX6A1/NDUFA11/ATP5MJ/COX6B1/SDHA/NDUFA13/ATP5F1D/NDUFB3/SLC25A6/CHCHD10
	CC	GO:0030672	synaptic vesicle membrane	8/376	114/19869	1.47E-03	4.86E-02	8	ATP6V0C/ATP6V1F/SYP/VAMP1/CALM3/RAB3A/ATP6V1E1/GABRA2
	CC	GO:0099501	exocytic vesicle membrane	8/376	114/19869	1.47E-03	4.86E-02	8	ATP6V0C/ATP6V1F/SYP/VAMP1/CALM3/RAB3A/ATP6V1E1/GABRA2
	CC	GO:0098798	mitochondrial protein-containing complex	14/376	295/19869	1.57E-03	4.86E-02	14	COX6A1/NDUFA11/ATP5MJ/COX6B1/SDHA/NDUFA13/IDH3B/IDH3G/MRPL4/ATP5F1D/NDUFB3/MRPS21/SLC25A6/CHCHD10
	MF	GO:0005200	structural constituent of cytoskeleton	10/363	107/18432	5.01E-05	3.23E-02	10	NEFH/TUBA1C/TUBA4A/TUBG2/TUBA8/SPTAN1/NEFM/ACTL6B/NEFL/TUBA1B
**Upregulated**	BP	GO:0060070	canonical Wnt signaling pathway	11/128	310/18903	8.57E-06	1.18E-02	11	EDN1/EDNRB/FGF2/SMURF2/ZNRF3/AMER2/UBR5/KLF4/TNKS2/SCYL2/LGR4
	BP	GO:0016055	Wnt signaling pathway	13/128	456/18903	1.35E-05	1.18E-02	13	EDN1/EDNRB/FGF2/CDC73/SMURF2/ZNRF3/AMER2/TNIK/UBR5/KLF4/TNKS2/SCYL2/LGR4
	BP	GO:0198738	cell-cell signaling by wnt	13/128	458/18903	1.41E-05	1.18E-02	13	EDN1/EDNRB/FGF2/CDC73/SMURF2/ZNRF3/AMER2/TNIK/UBR5/KLF4/TNKS2/SCYL2/LGR4
	BP	GO:0001738	morphogenesis of a polarized epithelium	6/128	96/18903	4.83E-05	2.84E-02	6	OPHN1/INTU/SMURF2/ZNRF3/NPHP1/LAMA1
	BP	GO:0072073	kidney epithelium development	7/128	146/18903	6.16E-05	2.84E-02	7	EDNRB/FGF2/HEYL/SMAD4/SDC4/LGR4/HES5

	BP	GO:0051216	cartilage development	8/128	201/18903	6.83E-05	2.84E-02	8	EDN1/FGF2/BMPR1B/NFIB/ITGB8/MBOAT2/BBS2/HES5
	BP	GO:0072006	nephron development	7/128	152/18903	7.94E-05	2.84E-02	7	EDNRB/FGF2/HEYL/SMAD4/COL4A3/LGR4/HES5
	BP	GO:0003018	vascular process in circulatory system	9/128	270/18903	9.39E-05	2.93E-02	9	EDN1/EDNRB/SLC1A2/SLC4A4/ADORA2B/BBS2/P2RY1/SLCO1C1/SLC15A2
	BP	GO:0061448	connective tissue development	9/128	274/18903	1.05E-04	2.93E-02	9	EDN1/FGF2/BMPR1B/NFIB/ITGB8/MBOAT2/BBS2/ID4/HES5
	BP	GO:0072009	nephron epithelium development	6/128	116/18903	1.38E-04	3.47E-02	6	EDNRB/FGF2/HEYL/SMAD4/LGR4/HES5
	BP	GO:0001655	urogenital system development	10/128	360/18903	1.72E-04	3.85E-02	10	EDNRB/FGF2/HEYL/SMAD4/COL4A3/NFIA/SDC4/ID4/LGR4/HES5
	BP	GO:0021953	central nervous system neuron differentiation	7/128	174/18903	1.84E-04	3.85E-02	7	OPHN1/BMPR1B/NFIB/ZSWIM6/SCYL2/ID4/HES5
**Downregulated**	BP	GO:0051588	regulation of neurotransmitter transport	10/231	105/18903	6.40E-07	6.70E-04	10	CPLX1/SNCG/FLOT1/SYP/STX1B/CALM3/RAB3A/VPS18/APBA2/CPLX2
	BP	GO:0050804	modulation of chemical synaptic transmission	20/231	448/18903	6.80E-07	6.70E-04	20	CPLX1/ARC/SNCG/FLOT1/VGF/NPAS4/NRGN/SYP/PTK2B/RASGRF1/STX1B/CALM3/EGR2/RAB3A/ADORA1/PAIP2/VPS18/APBA2/ARRB2/CPLX2
	BP	GO:0099177	regulation of trans-synaptic signaling	20/231	449/18903	7.04E-07	6.70E-04	20	CPLX1/ARC/SNCG/FLOT1/VGF/NPAS4/NRGN/SYP/PTK2B/RASGRF1/STX1B/CALM3/EGR2/RAB3A/ADORA1/PAIP2/VPS18/APBA2/ARRB2/CPLX2
	BP	GO:0046928	regulation of neurotransmitter secretion	9/231	91/18903	1.71E-06	1.22E-03	9	CPLX1/SNCG/SYP/STX1B/CALM3/RAB3A/VPS18/APBA2/CPLX2
	BP	GO:0009060	aerobic respiration	12/231	194/18903	4.94E-06	2.63E-03	12	COX6A1/NDUFA11/COX6B1/SDHA/NDUFA13/IDH3B/IDH3G/ATP5F1D/CS/NDUFB3/MDH2/CHCHD10

	BP	GO:0009141	nucleoside triphosphate metabolic process	14/231	267/18903	5.54E-06	2.63E-03	14	PKM/ATP6V0C/NDUFA11/ATP5MJ/TMSB4X/PGAM4/NME1/SDHA/NDUFA13/DCTPP1/ATP5F1D/ENO2/NDUFB3/ATP5IF1
	BP	GO:0009205	purine ribonucleoside triphosphate metabolic process	13/231	242/18903	9.28E-06	3.38E-03	13	PKM/ATP6V0C/NDUFA11/ATP5MJ/TMSB4X/PGAM4/NME1/SDHA/NDUFA13/ATP5F1D/ENO2/NDUFB3/ATP5IF1
	BP	GO:0048167	regulation of synaptic plasticity	12/231	207/18903	9.58E-06	3.38E-03	12	ARC/VGF/NPAS4/NRGN/SYP/PTK2B/RASGRF1/EGR2/RAB3A/ADORA1/PAIP2/CPLX2
	BP	GO:0009144	purine nucleoside triphosphate metabolic process	13/231	247/18903	1.15E-05	3.38E-03	13	PKM/ATP6V0C/NDUFA11/ATP5MJ/TMSB4X/PGAM4/NME1/SDHA/NDUFA13/ATP5F1D/ENO2/NDUFB3/ATP5IF1
	BP	GO:0009199	ribonucleoside triphosphate metabolic process	13/231	249/18903	1.26E-05	3.38E-03	13	PKM/ATP6V0C/NDUFA11/ATP5MJ/TMSB4X/PGAM4/NME1/SDHA/NDUFA13/ATP5F1D/ENO2/NDUFB3/ATP5IF1
	BP	GO:0009206	purine ribonucleoside triphosphate biosynthetic process	9/231	117/18903	1.37E-05	3.38E-03	9	ATP6V0C/NDUFA11/ATP5MJ/TMSB4X/NME1/SDHA/NDUFA13/ATP5F1D/NDUFB3
	BP	GO:0009145	purine nucleoside triphosphate biosynthetic process	9/231	118/18903	1.47E-05	3.38E-03	9	ATP6V0C/NDUFA11/ATP5MJ/TMSB4X/NME1/SDHA/NDUFA13/ATP5F1D/NDUFB3
	BP	GO:0046034	ATP metabolic process	12/231	217/18903	1.54E-05	3.38E-03	12	PKM/ATP6V0C/NDUFA11/ATP5MJ/TMSB4X/PGAM4/SDHA/NDUFA13/ATP5F1D/ENO2/NDUFB3/ATP5IF1
	BP	GO:0009201	ribonucleoside triphosphate biosynthetic process	9/231	123/18903	2.05E-05	4.17E-03	9	ATP6V0C/NDUFA11/ATP5MJ/TMSB4X/NME1/SDHA/NDUFA13/ATP5F1D/NDUFB3
	BP	GO:0050808	synapse organization	17/231	432/18903	2.47E-05	4.69E-03	17	SNCB/NEFH/ARC/SNCG/CFL1/PIN1/NPAS4/SPOCK2/RAB3A/PFN1/DCTN1/HAPLN4/CACNB1/SEZ6L2/NEFL/NRG1/CHCHD10
	BP	GO:0006099	tricarboxylic acid cycle	5/231	31/18903	3.42E-05	6.10E-03	5	SDHA/IDH3B/IDH3G/CS/MDH2
	BP	GO:0015986	proton motive force-driven ATP synthesis	7/231	76/18903	3.97E-05	6.24E-03	7	ATP6V0C/NDUFA11/ATP5MJ/SDHA/NDUFA13/ATP5F1D/NDUFB3

	BP	GO:0009142	nucleoside triphosphate biosynthetic process	9/231	134/18903	4.04E-05	6.24E-03	9	ATP6V0C/NDUFA11/ATP5MJ/TMSB4X/NME1/SDHA/NDUFA13/ATP5F1D/NDUFB3
	BP	GO:0045333	cellular respiration	12/231	240/18903	4.16E-05	6.24E-03	12	COX6A1/NDUFA11/COX6B1/SDHA/NDUFA13/IDH3B/IDH3G/ATP5F1D/CS/NDUFB3/MDH2/CHCHD10
	BP	GO:0006754	ATP biosynthetic process	8/231	106/18903	4.75E-05	6.78E-03	8	ATP6V0C/NDUFA11/ATP5MJ/TMSB4X/SDHA/NDUFA13/ATP5F1D/NDUFB3
	BP	GO:0099003	vesicle-mediated transport in synapse	11/231	212/18903	6.23E-05	8.01E-03	11	SNCB/CPLX1/ARC/SNCG/SYP/STX1B/CALM3/RAB3A/VPS18/APBA2/CPLX2
	BP	GO:0016079	synaptic vesicle exocytosis	8/231	111/18903	6.60E-05	8.01E-03	8	CPLX1/SYP/STX1B/CALM3/RAB3A/VPS18/APBA2/CPLX2
	BP	GO:1903320	regulation of protein modification by small protein conjugation or removal	12/231	252/18903	6.65E-05	8.01E-03	12	COPS7A/UBB/LIMK1/PIN1/GABARAP/GPS2/EGR1/PPIA/HUWE1/RPS15/OTUB1/ARRB2
	BP	GO:0048168	regulation of neuronal synaptic plasticity	6/231	57/18903	6.73E-05	8.01E-03	6	ARC/VGF/SYP/RASGRF1/EGR2/RAB3A
	BP	GO:0007269	neurotransmitter secretion	9/231	151/18903	1.02E-04	1.12E-02	9	CPLX1/SNCG/SYP/STX1B/CALM3/RAB3A/VPS18/APBA2/CPLX2
	BP	GO:0099643	signal release from synapse	9/231	151/18903	1.02E-04	1.12E-02	9	CPLX1/SNCG/SYP/STX1B/CALM3/RAB3A/VPS18/APBA2/CPLX2
	BP	GO:0098780	response to mitochondrial depolarisation	4/231	21/18903	1.10E-04	1.17E-02	4	CDC37/GPS2/HUWE1/ATP5IF1
	BP	GO:0099504	synaptic vesicle cycle	10/231	192/18903	1.30E-04	1.33E-02	10	SNCB/CPLX1/SNCG/SYP/STX1B/CALM3/RAB3A/VPS18/APBA2/CPLX2
	BP	GO:0051258	protein polymerization	12/231	281/18903	1.86E-04	1.83E-02	12	PTK2B/TMSB4X/CAPZB/TUBG2/CORO1A/PFN1/TUBGCP2/DCTN1/SPTAN1/VDAC2/FKBP4/NEFL

	BP	GO:1901214	regulation of neuron death	13/231	325/18903	1.92E-04	1.83E-02	13	AARS1/SNCB/UBB/SNCG/PTK2B/CHGA/ADORA1/CORO1A/ENO2/EGR1/HYOU1/NEFL/ARRB2
	BP	GO:0048499	synaptic vesicle membrane organization	4/231	25/18903	2.25E-04	2.00E-02	4	CPLX1/SYP/STX1B/RAB3A
	BP	GO:0062149	detection of stimulus involved in sensory perception of pain	4/231	25/18903	2.25E-04	2.00E-02	4	ADORA1/TMEM120A/KCNA1/ARRB2
	BP	GO:0015980	energy derivation by oxidation of organic compounds	13/231	333/18903	2.43E-04	2.09E-02	13	COX6A1/NDUFA11/COX6B1/SDHA/NDUFA13/IDH3B/IDH3G/PPP1CA/ATP5F1D/CS/NDUFB3/MDH2/CHCHD10
	BP	GO:0030705	cytoskeleton-dependent intracellular transport	10/231	208/18903	2.49E-04	2.09E-02	10	KIF5A/NEFH/UBB/F8A1/TUBA1C/KIF17/UCHL1/FLOT2/NEFL/TUBA1B
	BP	GO:0031396	regulation of protein ubiquitination	10/231	210/18903	2.69E-04	2.19E-02	10	UBB/LIMK1/PIN1/GABARAP/GPS2/PPIA/HUWE1/RPS15/OTUB1/ARRB2
	BP	GO:1902915	negative regulation of protein polyubiquitination	3/231	11/18903	2.76E-04	2.19E-02	3	GPS2/PPIA/OTUB1
	BP	GO:0006836	neurotransmitter transport	10/231	212/18903	2.90E-04	2.24E-02	10	CPLX1/SNCG/FLOT1/SYP/STX1B/CALM3/RAB3A/VPS18/APBA2/CPLX2
	BP	GO:0001505	regulation of neurotransmitter levels	10/231	219/18903	3.76E-04	2.82E-02	10	CPLX1/SNCG/FLOT1/SYP/STX1B/CALM3/RAB3A/VPS18/APBA2/CPLX2
	BP	GO:0036465	synaptic vesicle recycling	6/231	79/18903	4.12E-04	3.02E-02	6	SNCB/SNCG/SYP/STX1B/CALM3/RAB3A
	BP	GO:0006119	oxidative phosphorylation	8/231	147/18903	4.58E-04	3.13E-02	8	COX6A1/NDUFA11/COX6B1/SDHA/NDUFA13/ATP5F1D/NDUFB3/CHCHD10
	BP	GO:1902914	regulation of protein polyubiquitination	4/231	30/18903	4.64E-04	3.13E-02	4	GABARAP/GPS2/PPIA/OTUB1

	BP	GO:0098935	dendritic transport	3/231	13/18903	4.71E-04	3.13E-02	3	KIF5A/KIF17/FLOT2
	BP	GO:0032272	negative regulation of protein polymerization	6/231	81/18903	4.72E-04	3.13E-02	6	TMSB4X/CAPZB/PFN1/SPTAN1/VDAC2/FKBP4
	BP	GO:1903050	regulation of proteolysis involved in protein catabolic process	10/231	227/18903	4.98E-04	3.23E-02	10	RAD23A/UBB/F8A1/UBQLN4/USP5/PTK2B/DDA1/GABARAP/PSMC3/ATP5IF1
	BP	GO:0042176	regulation of protein catabolic process	13/231	361/18903	5.24E-04	3.33E-02	13	RAD23A/UBB/F8A1/UBQLN4/PIN1/USP5/DDA1/GABARAP/PSMC3/EEF1A2/NDUFA13/SNX3/NRG1
	BP	GO:0002931	response to ischemia	5/231	55/18903	5.52E-04	3.43E-02	5	PTK2B/UCHL1/PANX2/EGR1/HYOU1
	BP	GO:0031397	negative regulation of protein ubiquitination	6/231	84/18903	5.73E-04	3.46E-02	6	LIMK1/GPS2/PPIA/RPS15/OTUB1/ARRB2
	BP	GO:0072350	tricarboxylic acid metabolic process	3/231	14/18903	5.94E-04	3.46E-02	3	IDH3B/IDH3G/CS
	BP	GO:1904925	positive regulation of autophagy of mitochondrion in response to mitochondrial depolarization	3/231	14/18903	5.94E-04	3.46E-02	3	CDC37/HUWE1/ATP5IF1
	BP	GO:0070997	neuron death	13/231	368/18903	6.27E-04	3.58E-02	13	AARS1/SNCB/UBB/SNCG/PTK2B/CHGA/ADORA1/CORO1A/ENO2/EGR1/HYOU1/NEFL/ARRB2
	BP	GO:1904923	regulation of autophagy of mitochondrion in response to mitochondrial depolarization	3/231	15/18903	7.35E-04	4.12E-02	3	CDC37/HUWE1/ATP5IF1
	BP	GO:0019693	ribose phosphate metabolic process	15/231	475/18903	7.80E-04	4.21E-02	15	PKM/ATP6V0C/HINT1/NDUFA11/ATP5MJ/TMSB4X/PGAM4/NME1/SDHA/NDUFA13/ATP5F1D/ENO2/NDUFB3/ATP5IF1/TKT

	BP	GO:0046390	ribose phosphate biosynthetic process	10/231	241/18903	7.92E-04	4.21E-02	10	ATP6V0C/NDUFA11/ATP5MJ/TMSB4X/NME1/SDHA/NDUFA13/ATP5F1D/NDUFB3/TKT
	BP	GO:1902600	proton transmembrane transport	8/231	160/18903	8.00E-04	4.21E-02	8	ATP6V0C/ATP6V1F/COX6A1/TMSB4X/COX6B1/ATP1A1/ATP5F1D/ATP6V1E1
	BP	GO:0045055	regulated exocytosis	10/231	242/18903	8.17E-04	4.21E-02	10	CPLX1/SYP/STX1B/CALM3/CHGA/RAB3A/CORO1A/VPS18/APBA2/CPLX2
	BP	GO:2000300	regulation of synaptic vesicle exocytosis	5/231	60/18903	8.25E-04	4.21E-02	5	SYP/CALM3/RAB3A/VPS18/APBA2
	BP	GO:0032271	regulation of protein polymerization	9/231	201/18903	8.43E-04	4.22E-02	9	PTK2B/TMSB4X/CAPZB/CORO1A/PFN1/DCTN1/SPTAN1/VDAC2/FKBP4
	BP	GO:0097345	mitochondrial outer membrane permeabilization	4/231	36/18903	9.42E-04	4.63E-02	4	VDAC2/ATP5IF1/SLC25A6/CHCHD10
	BP	GO:0051656	establishment of organelle localization	14/231	437/18903	1.01E-03	4.90E-02	14	KIF5A/NEFH/UBB/F8A1/TRAPPC1/CFL1/CHGA/RAB3A/UCHL1/DCTN1/NUDC/RPS15/NEFL/CPLX2
	BP	GO:0006734	NADH metabolic process	4/231	37/18903	1.05E-03	4.98E-02	4	PKM/IDH3B/ENO2/MDH2
	CC	GO:0043025	neuronal cell body	25/241	497/19869	2.10E-09	9.09E-07	25	SST/KIF5A/SNCB/CPLX1/UBB/ARC/SNCG/GLRX5/VGF/NRGN/CKB/PTK2B/GDI1/ADORA1/PPP1CA/ROGDI/KCND3/ENO2/UCHL1/DCTN1/FKBP4/KCNA1/SEZ6L2/FXR2/CPLX2
	CC	GO:0150034	distal axon	18/241	278/19869	9.15E-09	1.98E-06	18	SNCB/CPLX1/SNCG/CFL1/SYP/PTK2B/RASGRF1/CALM3/RAB3A/ADORA1/ENO2/COPA/TAOK2/FKBP4/KCNA1/NEFL/FXR2/CPLX2
	CC	GO:0098798	mitochondrial protein-containing complex	14/241	295/19869	1.59E-05	2.30E-03	14	COX6A1/NDUFA11/ATP5MJ/COX6B1/SDHA/NDUFA13/IDH3B/IDH3G/MRPL4/ATP5F1D/NDUFB3/MRPS21/SLC25A6/CHCHD10
	CC	GO:0098800	inner mitochondrial membrane protein complex	10/241	158/19869	2.38E-05	2.58E-03	10	COX6A1/NDUFA11/ATP5MJ/COX6B1/SDHA/NDUFA13/ATP5F1D/NDUFB3/SLC25A6/CHCHD10

	CC	GO:0044306	neuron projection terminus	9/241	131/19869	3.20E-05	2.77E-03	9	SNCB/CPLX1/SNCG/SYP/RAB3A/ADORA1/UCHL1/KCNA1/CPLX2
	CC	GO:0030426	growth cone	10/241	167/19869	3.83E-05	2.77E-03	10	CFL1/PTK2B/RASGRF1/CALM3/ENO2/COPA/TAOK2/FKBP4/NEFL/FXR2
	CC	GO:0030427	site of polarized growth	10/241	173/19869	5.17E-05	3.17E-03	10	CFL1/PTK2B/RASGRF1/CALM3/ENO2/COPA/TAOK2/FKBP4/NEFL/FXR2
	CC	GO:0005874	microtubule	17/241	466/19869	5.86E-05	3.17E-03	17	KIF5A/TUBA1C/TBCB/GABARAP/KIF17/TUBA4A/CALM3/TUBG2/TUBA8/KLC2/TUBGCP2/DCTN1/NUDC/DYNLRB1/CCT7/FKBP4/TUBA1B
	CC	GO:0043679	axon terminus	8/241	115/19869	8.08E-05	3.89E-03	8	SNCB/CPLX1/SNCG/SYP/RAB3A/ADORA1/KCNA1/CPLX2
	CC	GO:0098978	glutamatergic synapse	13/241	324/19869	1.74E-04	7.55E-03	13	CPLX1/ARC/FLOT1/PIN1/NRGN/PTK2B/PPP1CA/CORO1A/PFN1/VPS18/FLOT2/KCNA1/NRG1
	CC	GO:0008021	synaptic vesicle	10/241	208/19869	2.36E-04	8.49E-03	10	ATP6V0C/ATP6V1F/SYP/VAMP1/STX1B/CALM3/RAB3A/ROGDI/APBA2/ATP6V1E1
	CC	GO:0098685	Schaffer collateral - CA1 synapse	6/241	72/19869	2.40E-04	8.49E-03	6	NEFH/CPLX1/SYP/CAPZB/APBA2/NEFL
	CC	GO:0043195	terminal bouton	5/241	47/19869	2.55E-04	8.49E-03	5	CPLX1/SYP/RAB3A/ADORA1/CPLX2
	CC	GO:0043197	dendritic spine	9/241	175/19869	2.95E-04	8.87E-03	9	ARC/NRGN/PTK2B/ADORA1/PPP1CA/KCND3/APBA2/FXR2/ARRB2
	CC	GO:0044309	neuron spine	9/241	176/19869	3.07E-04	8.87E-03	9	ARC/NRGN/PTK2B/ADORA1/PPP1CA/KCND3/APBA2/FXR2/ARRB2
	CC	GO:0016469	proton-transporting two-sector ATPase complex	5/241	50/19869	3.42E-04	9.25E-03	5	ATP6V0C/ATP6V1F/ATP5MJ/ATP5F1D/ATP6V1E1

	CC	GO:0005938	cell cortex	12/241	312/19869	4.52E-04	1.05E-02	12	ARC/FLOT1/FMNL1/PTK2B/CAPZB/CORO1A/ENO2/PFN1/DCTN1/FLOT2/SPTAN1/CABP1
	CC	GO:0070382	exocytic vesicle	10/241	226/19869	4.57E-04	1.05E-02	10	ATP6V0C/ATP6V1F/SYP/VAMP1/STX1B/CALM3/RAB3A/ROGDI/APBA2/ATP6V1E1
	CC	GO:0030672	synaptic vesicle membrane	7/241	114/19869	4.85E-04	1.05E-02	7	ATP6V0C/ATP6V1F/SYP/VAMP1/CALM3/RAB3A/ATP6V1E1
	CC	GO:0099501	exocytic vesicle membrane	7/241	114/19869	4.85E-04	1.05E-02	7	ATP6V0C/ATP6V1F/SYP/VAMP1/CALM3/RAB3A/ATP6V1E1
	CC	GO:0014069	postsynaptic density	12/241	321/19869	5.81E-04	1.20E-02	12	NEFH/ARC/NRGN/PTK2B/GNG3/ATP1A1/ADORA1/CAPZB/KCND3/FXR2/ARRB2/CABP1
	CC	GO:0005925	focal adhesion	14/241	422/19869	6.78E-04	1.25E-02	14	ATP6V0C/FLOT1/LIMK1/CFL1/PTK2B/PDLIM7/SPRY4/CORO2B/PFN1/RPL18/PPIA/FLOT2/HYOU1/RPS15
	CC	GO:0032279	asymmetric synapse	12/241	327/19869	6.84E-04	1.25E-02	12	NEFH/ARC/NRGN/PTK2B/GNG3/ATP1A1/ADORA1/CAPZB/KCND3/FXR2/ARRB2/CABP1
	CC	GO:0030133	transport vesicle	14/241	423/19869	6.94E-04	1.25E-02	14	ATP6V0C/ATP6V1F/VGF/NRGN/SYP/VAMP1/STX1B/CALM3/CHGA/RAB3A/ROGDI/COPA/APBA2/ATP6V1E1
	CC	GO:0120111	neuron projection cytoplasm	6/241	90/19869	7.97E-04	1.36E-02	6	KIF5A/ARC/KIF17/UCHL1/FLOT2/NEFL
	CC	GO:0098803	respiratory chain complex	6/241	91/19869	8.45E-04	1.36E-02	6	COX6A1/NDUFA11/COX6B1/SDHA/NDUFA13/NDUFB3
	CC	GO:0030055	cell-substrate junction	14/241	432/19869	8.51E-04	1.36E-02	14	ATP6V0C/FLOT1/LIMK1/CFL1/PTK2B/PDLIM7/SPRY4/CORO2B/PFN1/RPL18/PPIA/FLOT2/HYOU1/RPS15
	CC	GO:0005746	mitochondrial respirasome	6/241	94/19869	1.00E-03	1.51E-02	6	COX6A1/NDUFA11/COX6B1/SDHA/NDUFA13/NDUFB3

	CC	GO:0099572	postsynaptic specialization	12/241	342/19869	1.01E-03	1.51E-02	12	NEFH/ARC/NRGN/PTK2B/GNG3/ATP1A1/ADORA1/CAPZB/KCND3/FXR2/ARRB2/CABP1
	CC	GO:0044305	calyx of Held	3/241	18/19869	1.26E-03	1.76E-02	3	CPLX1/ADORA1/KCNA1
	CC	GO:0098984	neuron to neuron synapse	12/241	351/19869	1.26E-03	1.76E-02	12	NEFH/ARC/NRGN/PTK2B/GNG3/ATP1A1/ADORA1/CAPZB/KCND3/FXR2/ARRB2/CABP1
	CC	GO:0070469	respirasome	6/241	101/19869	1.45E-03	1.94E-02	6	COX6A1/NDUFA11/COX6B1/SDHA/NDUFA13/NDUFB3
	CC	GO:0033178	proton-transporting two-sector ATPase complex, catalytic domain	3/241	19/19869	1.48E-03	1.94E-02	3	ATP6V1F/ATP5F1D/ATP6V1E1
	CC	GO:0048786	presynaptic active zone	5/241	75/19869	2.18E-03	2.78E-02	5	FLOT1/SYP/STX1B/RAB3A/ADORA1
	CC	GO:0005759	mitochondrial matrix	14/241	483/19869	2.41E-03	2.99E-02	14	GLRX5/PRDX5/IDH3B/IDH3G/MRPL4/ATP5F1D/CS/MDH2/VDAC2/MYG1/TUFM/MRPS21/NAT8L/NAXE
	CC	GO:0031201	SNARE complex	4/241	48/19869	2.70E-03	3.25E-02	4	CPLX1/VAMP1/STX1B/CPLX2
	CC	GO:0043204	perikaryon	7/241	154/19869	2.78E-03	3.26E-02	7	KIF5A/CPLX1/PPP1CA/ROGDI/ENO2/KCNA1/CPLX2
	CC	GO:0016471	vacuolar proton-transporting V-type ATPase complex	3/241	25/19869	3.33E-03	3.79E-02	3	ATP6V0C/ATP6V1F/ATP6V1E1
	CC	GO:0033176	proton-transporting V-type ATPase complex	3/241	27/19869	4.16E-03	4.47E-02	3	ATP6V0C/ATP6V1F/ATP6V1E1
	CC	GO:0005875	microtubule associated complex	7/241	166/19869	4.21E-03	4.47E-02	7	KIF5A/GABARAP/KIF17/KLC2/DCTN1/DCTN3/DYNLRB1

	CC	GO:1990204	oxidoreductase complex	6/241	125/19869	4.23E-03	4.47E-02	6	NDUFA11/SDHA/NDUFA13/ IDH3B/IDH3G/NDUFB3
	MF	GO:0005200	structural constituent of cytoskeleton	10/234	107/18432	1.08E-06	5.21E-04	10	NEFH/TUBA1C/TUBA4A/TUBG2/TUBA8/ SPTAN1/NEFM/ACTL6B/NEFL/TUBA1B

Supplementary Table 4. Functional annotation of the 28 coexpression modules differentially expressed between samples from subjects with antisocial personality disorder (ASPD) and/or conduct disorder (CD) vs unaffected controls. Only the top Gene Ontology enriched classes are reported.

module	Module parent	log2 FC	P.Value	adj.P.Val	Size	GO (Cellular component)	GO (Molecular function)	GO (Biological process)	Hub	Cell Enrichment
M61	M7	-0.482	1.5E-04	2.9E-02	14				*UBQLN4(7)*	-
M148	M20	-0.464	2.6E-04	2.9E-02	12					-
M63	M7	0.449	4.1E-04	2.9E-02	25				*INTU(11)*	-
M237	M34	-0.445	4.5E-04	2.9E-02	16				*FARSA(11)*	-
M286	M65	0.443	4.8E-04	2.9E-02	14				*FIBP(11)*	-
M111	M17	-0.436	5.9E-04	3.0E-02	51				*SST(14)*	-
M65	M7	-0.425	8.1E-04	3.0E-02	21				*FIBP(14)*	-
M7	M1	-0.421	9.0E-04	3.0E-02	307	postsynaptic specialization	synaptic vesicle recycling		*CALM3(23)*	-
M38	M2	0.414	1.1E-03	3.0E-02	11					-
M565	M373	-0.413	1.1E-03	3.0E-02	16				*TRIR(10)*	-
M372	M129	-0.409	1.3E-03	3.0E-02	27			protein hetero-dimerization activity	*UBB(13)*	-
M373	M129	-0.409	1.3E-03	3.0E-02	31				*TRIR(13)*	-
M18	M1	-0.408	1.3E-03	3.0E-02	74	ribosomal subunit			*TRIR(18)*	-
M129	M18	-0.404	1.4E-03	3.0E-02	70	ribosomal subunit			*TRIR(18)*	-
M353	M111	-0.404	1.5E-03	3.0E-02	13				*CAPNS1(9)*	-
M34	M2	-0.399	1.7E-03	3.1E-02	72	transport vesicle			*RAD23A(24)*	-
M2	M1	-0.399	1.7E-03	3.1E-02	151				*RAD23A(25)*	-
M234	M34	-0.394	1.9E-03	3.2E-02	23				*GDI1(12)*	-
M589	M399	0.391	2.1E-03	3.3E-02	13				*CPNE9(11)*	-
M59	M7	-0.386	2.4E-03	3.5E-02	23				*CPLX1(14)*	-
M228	M31	-0.384	2.5E-03	3.5E-02	29	mitochondrial inner membrane	cellular respiration		*EIF6(10)*	-
M277	M58	0.383	2.5E-03	3.5E-02	11				*SMAP2(7)*	-

M275	M58	-0.378	2.9E-03	3.9E-02	13				*APBA2(10)*	-
M384	M132	-0.376	3.0E-03	3.9E-02	11				*PRDX5(7)*	-
M441	M182	0.369	3.6E-03	4.3E-02	17				*PTTG1IP(11)*	-
M144	M20	0.368	3.7E-03	4.3E-02	31				*AGL(11)*	Ast
M402	M144	0.367	3.8E-03	4.3E-02	17				*AGL(10)*	Ast

Supplementary Table 5. Complete results of the GO analyses by coexpression module. (Only modules differentially expressed between ASPD+CD vs CTL have been analyzed).

Module	Ontology	ID	Description	p	Adjust. p	Count	geneID
M7	CC	GO:0099572	postsynaptic specialization	1.62E-05	4.97E-03	17	*ADORA1/ATP2B2/BCL11A/CABP1/CACNG3/DMTN/FXR2/GABRA2/GNG3/GRIN2D/IQSEC3/KCND1/LRRC4/P2RX6/PPP1R9B/SIGMAR1/SYN1*
	CC	GO:0014069	postsynaptic density	2.78E-05	4.97E-03	16	*ADORA1/ATP2B2/BCL11A/CABP1/CACNG3/DMTN/FXR2/GNG3/GRIN2D/IQSEC3/KCND1/LRRC4/P2RX6/PPP1R9B/SIGMAR1/SYN1*
	CC	GO:0032279	asymmetric synapse	3.47E-05	4.97E-03	16	*ADORA1/ATP2B2/BCL11A/CABP1/CACNG3/DMTN/FXR2/GNG3/GRIN2D/IQSEC3/KCND1/LRRC4/P2RX6/PPP1R9B/SIGMAR1/SYN1*
	CC	GO:0098984	neuron to neuron synapse	8.04E-05	8.63E-03	16	*ADORA1/ATP2B2/BCL11A/CABP1/CACNG3/DMTN/FXR2/GNG3/GRIN2D/IQSEC3/KCND1/LRRC4/P2RX6/PPP1R9B/SIGMAR1/SYN1*
	CC	GO:0097060	synaptic membrane	1.89E-04	1.62E-02	16	*ADAM23/ADORA1/ATP2B2/CACNG3/DNM1/EPHB2/GABRA2/GRIN2D/IQSEC3/KCND1/LRRC4/P2RX6/PPP1R9B/SIGMAR1/STX1B/UNC13A*
	CC	GO:0150034	distal axon	2.91E-04	1.71E-02	13	*ADORA1/BASP1/CALM3/CDK5R2/CFL1/CPLX1/FXR2/HCN3/OPHN1/PPP1R9B/SIGMAR1/SNCB/UNC13A*
	CC	GO:0043197	dendritic spine	3.05E-04	1.71E-02	10	*ADORA1/APBA2/ATP2B2/EPHB2/FXR2/KCND1/LRRC4/OPHN1/P2RX6/PPP1R9B*
	CC	GO:0044309	neuron spine	3.19E-04	1.71E-02	10	*ADORA1/APBA2/ATP2B2/EPHB2/FXR2/KCND1/LRRC4/OPHN1/P2RX6/PPP1R9B*
	CC	GO:0099055	integral component of postsynaptic membrane	3.92E-04	1.74E-02	8	*ADORA1/ATP2B2/CACNG3/EPHB2/GABRA2/GRIN2D/LRRC4/P2RX6*
	CC	GO:0098936	intrinsic component of postsynaptic membrane	5.17E-04	1.93E-02	8	*ADORA1/ATP2B2/CACNG3/EPHB2/GABRA2/GRIN2D/LRRC4/P2RX6*
	CC	GO:0099699	integral component of synaptic membrane	5.40E-04	1.93E-02	9	*ADAM23/ADORA1/ATP2B2/CACNG3/EPHB2/GABRA2/GRIN2D/LRRC4/P2RX6*
	CC	GO:0098685	Schaffer collateral - CA1 synapse	7.12E-04	2.35E-02	6	*APBA2/CACNG3/CPLX1/LRRC4/PLAT/SYN1*
	CC	GO:0099240	intrinsic component of synaptic membrane	8.82E-04	2.52E-02	9	*ADAM23/ADORA1/ATP2B2/CACNG3/EPHB2/GABRA2/GRIN2D/LRRC4/P2RX6*
	CC	GO:0099060	integral component of postsynaptic specialization membrane	9.48E-04	2.54E-02	6	*ATP2B2/CACNG3/GABRA2/GRIN2D/LRRC4/P2RX6*
	CC	GO:0098948	intrinsic component of postsynaptic specialization membrane	1.16E-03	2.74E-02	6	*ATP2B2/CACNG3/GABRA2/GRIN2D/LRRC4/P2RX6*
	CC	GO:0008021	synaptic vesicle	1.17E-03	2.74E-02	10	*APBA2/ATP6V1F/CALM3/GABRA2/LGI3/RAB35/STX1B/SYN1/SYT5/UNC13A*
	CC	GO:0098978	glutamatergic synapse	1.21E-03	2.74E-02	13	*ADAM23/ARHGAP39/ATP2B2/CACNG3/CPLX1/DNM1/EPHB2/GRIN2D/LRRC4/OPHN1/P2RX6/PIN1/PLAT*
	CC	GO:0030672	synaptic vesicle membrane	1.61E-03	3.28E-02	7	*ATP6V1F/CALM3/GABRA2/RAB35/SYN1/SYT5/UNC13A*
	CC	GO:0099501	exocytic vesicle membrane	1.61E-03	3.28E-02	7	*ATP6V1F/CALM3/GABRA2/RAB35/SYN1/SYT5/UNC13A*
	CC	GO:0099634	postsynaptic specialization membrane	1.69E-03	3.29E-02	7	*ATP2B2/CACNG3/GABRA2/GRIN2D/LRRC4/P2RX6/SIGMAR1*
	CC	GO:0070382	exocytic vesicle	2.16E-03	3.86E-02	10	*APBA2/ATP6V1F/CALM3/GABRA2/LGI3/RAB35/STX1B/SYN1/SYT5/UNC13A*

	CC	GO:0045211	postsynaptic membrane	2.45E-03	4.20E-02	11	*ADORA1/ATP2B2/CACNG3/EPHB2/GABRA2/ GRIN2D/IQSEC3/KCND1/LRRC4/P2RX6/SIGMAR1*
	BP	GO:0036465	synaptic vesicle recycling	2.15E-05	4.79E-02	8	*AP3B1/CALM3/DNM1/OPHN1/ROCK1/SNCB/STX1B/SYT5*
	BP	GO:0099504	synaptic vesicle cycle	2.95E-05	4.79E-02	12	*AP3B1/APBA2/CALM3/CPLX1/DNM1/OPHN1/ ROCK1/SNCB/STX1B/SYN1/SYT5/UNC13A*
M372	MF	GO:0046982	protein heterodimerization activity	8.25E-05	1.01E-02	5	*FZD4/H4C15/H4C11/H4C12/SUPT5H*
M34	CC	GO:0030133	transport vesicle	9.56E-05	1.84E-02	8	*ATP6V0C/CAV2/COPB1/NRGN/RAB3A/ROGDI/RPH3A/YIPF2*
M228	BP	GO:0045333	cellular respiration	1.46E-06	7.90E-04	6	*COX5B/NDUFV1/NOA1/NOP53/OGDHL/SURF1*
	BP	GO:0015980	energy derivation by oxidation of organic compounds	9.64E-06	1.89E-03	6	*COX5B/NDUFV1/NOA1/NOP53/OGDHL/SURF1*
	BP	GO:0009060	aerobic respiration	1.05E-05	1.89E-03	5	*COX5B/NDUFV1/NOP53/OGDHL/SURF1*
	BP	GO:0006364	rRNA processing	2.34E-05	3.16E-03	5	*EIF6/NOP53/RPL27/RPS15/RPUSD1*
	BP	GO:0016072	rRNA metabolic process	4.96E-05	5.36E-03	5	*EIF6/NOP53/RPL27/RPS15/RPUSD1*
	BP	GO:0042254	ribosome biogenesis	9.87E-05	8.49E-03	5	*EIF6/NOP53/RPL27/RPS15/RPUSD1*
	CC	GO:0005743	mitochondrial inner membrane	7.00E-04	9.80E-03	5	*COX5B/MRPL14/NDUFV1/NOA1/SURF1*
	BP	GO:0034470	ncRNA processing	4.95E-04	2.67E-02	5	*EIF6/NOP53/RPL27/RPS15/RPUSD1*
	BP	GO:0022613	ribonucleoprotein complex biogenesis	8.06E-04	3.96E-02	5	*EIF6/NOP53/RPL27/RPS15/RPUSD1*
M18	CC	GO:0044391	ribosomal subunit	3.75E-04	1.20E-02	5	*GADD45GIP1/MRPS18B/MRPS34/RACK1/RPS28*
	CC	GO:0005840	ribosome	9.71E-04	2.59E-02	5	*GADD45GIP1/MRPS18B/MRPS34/RACK1/RPS28*
M129	CC	GO:0044391	ribosomal subunit	2.78E-04	8.69E-03	5	*GADD45GIP1/MRPS18B/MRPS34/RACK1/RPS28*
	CC	GO:0005840	ribosome	7.26E-04	1.89E-02	5	*GADD45GIP1/MRPS18B/MRPS34/RACK1/RPS28*

Supplementary Table 6. Complete cell enrichment results for the significantly different coexpression modules between antisocial personality disorder (ASPD)+ conduct disorder (CD) vs unaffected controls. In bold and red, the significant enrichments results (FDR < 0.05).

Module	Cell type	Genes	pval	padj
M2	End	6	1.2E-01	9.7E-01
	Ex	16	3.7E-01	1.0E+00
	Ast	6	8.4E-01	1.0E+00
	Opc	2	8.7E-01	1.0E+00
	Mic	3	9.1E-01	1.0E+00
	In	1	9.3E-01	1.0E+00
	Oli	1	9.9E-01	1.0E+00
	Per	0	1.0E+00	1.0E+00
M7	Ex	36	1.1E-01	9.2E-01
	Opc	9	2.7E-01	1.0E+00
	Per	4	6.2E-01	1.0E+00
	In	4	7.9E-01	1.0E+00
	Ast	14	8.0E-01	1.0E+00
	End	4	9.2E-01	1.0E+00
	Mic	5	9.9E-01	1.0E+00
	Oli	3	1.0E+00	1.0E+00
M18	In	2	3.7E-01	1.0E+00
	Ast	4	5.8E-01	1.0E+00
	Oli	2	7.2E-01	1.0E+00
	End	1	8.1E-01	1.0E+00
	Opc	1	8.2E-01	1.0E+00
	Mic	1	9.3E-01	1.0E+00
	Ex	4	9.3E-01	1.0E+00
	Per	0	1.0E+00	1.0E+00
M34	Ex	8	3.9E-01	1.0E+00
	End	2	4.8E-01	1.0E+00
	Opc	2	5.0E-01	1.0E+00
	In	1	7.2E-01	1.0E+00
	Ast	2	9.1E-01	1.0E+00
	Mic	0	1.0E+00	1.0E+00
	Oli	0	1.0E+00	1.0E+00
	Per	0	1.0E+00	1.0E+00
M38	Ex	2	2.8E-01	1.0E+00
	Ast	0	1.0E+00	1.0E+00
	Mic	0	1.0E+00	1.0E+00
	In	0	1.0E+00	1.0E+00
	Oli	0	1.0E+00	1.0E+00
	Per	0	1.0E+00	1.0E+00
	End	0	1.0E+00	1.0E+00
	Opc	0	1.0E+00	1.0E+00

M59	Ex	3	3.8E-01	1.0E+00
	Ast	0	1.0E+00	1.0E+00
	Mic	0	1.0E+00	1.0E+00
	In	0	1.0E+00	1.0E+00
	Oli	0	1.0E+00	1.0E+00
	Per	0	1.0E+00	1.0E+00
	End	0	1.0E+00	1.0E+00
	Opc	0	1.0E+00	1.0E+00
M61	Ex	3	1.4E-01	7.0E-01
	Per	1	1.8E-01	7.0E-01
	End	1	2.7E-01	7.2E-01
	Ast	0	1.0E+00	1.0E+00
	Mic	0	1.0E+00	1.0E+00
	In	0	1.0E+00	1.0E+00
	Oli	0	1.0E+00	1.0E+00
	Opc	0	1.0E+00	1.0E+00
M63	Ast	5	1.0E-02	8.1E-02
	Oli	1	5.8E-01	1.0E+00
	Ex	0	1.0E+00	1.0E+00
	Mic	0	1.0E+00	1.0E+00
	In	0	1.0E+00	1.0E+00
	Per	0	1.0E+00	1.0E+00
	End	0	1.0E+00	1.0E+00
	Opc	0	1.0E+00	1.0E+00
M65	Ast	2	3.2E-01	1.0E+00
	Opc	1	3.9E-01	1.0E+00
	Ex	2	6.1E-01	1.0E+00
	Mic	0	1.0E+00	1.0E+00
	In	0	1.0E+00	1.0E+00
	Oli	0	1.0E+00	1.0E+00
	Per	0	1.0E+00	1.0E+00
	End	0	1.0E+00	1.0E+00
M111	End	2	3.2E-01	1.0E+00
	Ex	5	5.5E-01	1.0E+00
	In	1	5.9E-01	1.0E+00
	Opc	1	7.0E-01	1.0E+00
	Ast	0	1.0E+00	1.0E+00
	Mic	0	1.0E+00	1.0E+00
	Oli	0	1.0E+00	1.0E+00
	Per	0	1.0E+00	1.0E+00
M129	In	2	3.4E-01	1.0E+00
	Ast	4	5.4E-01	1.0E+00
	Oli	2	6.9E-01	1.0E+00
	End	1	8.0E-01	1.0E+00
	Opc	1	8.1E-01	1.0E+00
	Ex	4	9.1E-01	1.0E+00
	Mic	1	9.2E-01	1.0E+00
	Per	0	1.0E+00	1.0E+00

M144	**Ast**	**9**	**2.7E-05**	**2.1E-04**
	Opc	1	5.1E-01	1.0E+00
	Mic	1	6.8E-01	1.0E+00
	Ex	1	9.6E-01	1.0E+00
	In	0	1.0E+00	1.0E+00
	Oli	0	1.0E+00	1.0E+00
	Per	0	1.0E+00	1.0E+00
	End	0	1.0E+00	1.0E+00
M148	Ex	1	7.0E-01	1.0E+00
	Ast	0	1.0E+00	1.0E+00
	Mic	0	1.0E+00	1.0E+00
	In	0	1.0E+00	1.0E+00
	Oli	0	1.0E+00	1.0E+00
	Per	0	1.0E+00	1.0E+00
	End	0	1.0E+00	1.0E+00
	Opc	0	1.0E+00	1.0E+00
M228	Ex	5	1.4E-01	1.0E+00
	End	1	4.8E-01	1.0E+00
	Opc	1	4.9E-01	1.0E+00
	Ast	1	8.0E-01	1.0E+00
	Mic	0	1.0E+00	1.0E+00
	In	0	1.0E+00	1.0E+00
	Oli	0	1.0E+00	1.0E+00
	Per	0	1.0E+00	1.0E+00
M234	Ex	5	6.3E-02	5.0E-01
	In	1	3.3E-01	9.6E-01
	Ast	2	3.6E-01	9.6E-01
	Mic	0	1.0E+00	1.0E+00
	Oli	0	1.0E+00	1.0E+00
	Per	0	1.0E+00	1.0E+00
	End	0	1.0E+00	1.0E+00
	Opc	0	1.0E+00	1.0E+00
M237	Opc	1	3.1E-01	1.0E+00
	Ex	2	4.6E-01	1.0E+00
	Ast	0	1.0E+00	1.0E+00
	Mic	0	1.0E+00	1.0E+00
	In	0	1.0E+00	1.0E+00
	Oli	0	1.0E+00	1.0E+00
	Per	0	1.0E+00	1.0E+00
	End	0	1.0E+00	1.0E+00
M275	Ex	2	3.6E-01	1.0E+00
	Ast	0	1.0E+00	1.0E+00
	Mic	0	1.0E+00	1.0E+00
	In	0	1.0E+00	1.0E+00
	Oli	0	1.0E+00	1.0E+00
	Per	0	1.0E+00	1.0E+00
	End	0	1.0E+00	1.0E+00
	Opc	0	1.0E+00	1.0E+00

M277	Per	2	9.5E-03	7.6E-02
	Ex	2	2.8E-01	8.8E-01
	Mic	1	3.3E-01	8.8E-01
	Ast	0	1.0E+00	1.0E+00
	In	0	1.0E+00	1.0E+00
	Oli	0	1.0E+00	1.0E+00
	End	0	1.0E+00	1.0E+00
	Opc	0	1.0E+00	1.0E+00
M286	Ast	2	1.8E-01	1.0E+00
	Opc	1	2.8E-01	1.0E+00
	Ex	0	1.0E+00	1.0E+00
	Mic	0	1.0E+00	1.0E+00
	In	0	1.0E+00	1.0E+00
	Oli	0	1.0E+00	1.0E+00
	Per	0	1.0E+00	1.0E+00
	End	0	1.0E+00	1.0E+00
M353	In	1	2.0E-01	1.0E+00
	Ex	2	3.6E-01	1.0E+00
	Ast	0	1.0E+00	1.0E+00
	Mic	0	1.0E+00	1.0E+00
	Oli	0	1.0E+00	1.0E+00
	Per	0	1.0E+00	1.0E+00
	End	0	1.0E+00	1.0E+00
	Opc	0	1.0E+00	1.0E+00
M372	Ex	4	2.6E-01	9.2E-01
	In	1	3.8E-01	9.2E-01
	Ast	2	4.4E-01	9.2E-01
	End	1	4.6E-01	9.2E-01
	Oli	1	6.1E-01	9.7E-01
	Mic	0	1.0E+00	1.0E+00
	Per	0	1.0E+00	1.0E+00
	Opc	0	1.0E+00	1.0E+00
M373	In	1	4.2E-01	1.0E+00
	Oli	1	6.6E-01	1.0E+00
	Mic	1	6.8E-01	1.0E+00
	Ast	1	8.3E-01	1.0E+00
	Ex	0	1.0E+00	1.0E+00
	Per	0	1.0E+00	1.0E+00
	End	0	1.0E+00	1.0E+00
	Opc	0	1.0E+00	1.0E+00
M384	Ex	2	2.8E-01	1.0E+00
	Mic	1	3.3E-01	1.0E+00
	Ast	1	4.6E-01	1.0E+00
	In	0	1.0E+00	1.0E+00
	Oli	0	1.0E+00	1.0E+00
	Per	0	1.0E+00	1.0E+00
	End	0	1.0E+00	1.0E+00
	Opc	0	1.0E+00	1.0E+00

M402	**Ast**	**6**	**1.9E-04**	**1.5E-03**
	Mic	1	4.6E-01	1.0E+00
	Ex	0	1.0E+00	1.0E+00
	In	0	1.0E+00	1.0E+00
	Oli	0	1.0E+00	1.0E+00
	Per	0	1.0E+00	1.0E+00
	End	0	1.0E+00	1.0E+00
	Opc	0	1.0E+00	1.0E+00
M441	Per	1	2.1E-01	8.5E-01
	Ex	3	2.2E-01	8.5E-01
	End	1	3.2E-01	8.5E-01
	Mic	1	4.6E-01	9.2E-01
	Ast	0	1.0E+00	1.0E+00
	In	0	1.0E+00	1.0E+00
	Oli	0	1.0E+00	1.0E+00
	Opc	0	1.0E+00	1.0E+00
M565	In	1	2.4E-01	1.0E+00
	Oli	1	4.2E-01	1.0E+00
	Mic	1	4.4E-01	1.0E+00
	Ast	0	1.0E+00	1.0E+00
	Ex	0	1.0E+00	1.0E+00
	Per	0	1.0E+00	1.0E+00
	End	0	1.0E+00	1.0E+00
	Opc	0	1.0E+00	1.0E+00
M589	Ast	2	1.6E-01	1.0E+00
	Opc	1	2.6E-01	1.0E+00
	Ex	1	7.3E-01	1.0E+00
	Mic	0	1.0E+00	1.0E+00
	In	0	1.0E+00	1.0E+00
	Oli	0	1.0E+00	1.0E+00
	Per	0	1.0E+00	1.0E+00
	End	0	1.0E+00	1.0E+00
